# Boosting CO_2_ hydrogenation to C_2+_ hydrocarbons *via* phase modulation using a Ni-doped CuCo catalyst

**DOI:** 10.1039/d6sc05518d

**Published:** 2026-09-29

**Authors:** Chenglong Yu, Jun He, Longbo Zhang, Ying Wang, Xianyu Meng, Yanru Zhang, Yanyan Wang, Jia Guo, Zhuo Zhang, Xinning Song, Bin Zhang, Qingli Qian, Buxing Han

**Affiliations:** a Beijing National Laboratory for Molecular Sciences, CAS Key Laboratory of Colloid and Interface and Thermodynamics, CAS Research/Education Center for Excellence in Molecular Sciences, Center for Carbon Neutral Chemistry, Institute of Chemistry, Chinese Academy of Sciences Beijing 100190 China qianql@iccas.ac.cn hanbx@iccas.ac.cn; b School of Chemical Sciences, University of Chinese Academy of Sciences Beijing 100049 China; c College of Chemical Engineering and Materials Science, Tianjin University of Science and Technology Tianjin 300457 China

## Abstract

The catalytic hydrogenation of CO_2_ to C_2+_ hydrocarbons is one of the most promising routes to achieving carbon neutrality. However, the development of effective catalysts to promote this transformation is still a challenging task. This work modulates the phase composition of CuCo bimetallic oxide catalysts through Ni doping, thereby influencing the strong electronic interactions at the material's surface and interface. The Ni doping promotes the production of straight-chain C_2+_ hydrocarbons *via* CO_2_ hydrogenation, without formation of oxygenates. Notably, the Ni_0.1_Cu_1_/CoO_*x*_ catalyst with Ni doping at a Ni : Cu ratio of 0.1 exhibits an impressive 63.0% selectivity for C_2+_ hydrocarbons during liquid–solid phase CO_2_ hydrogenation at a low temperature of 200 °C. With the phase transformation during the Ni doping, the strong interactions between Cu oxide and Co oxide species are enhanced, and the electronic structures of Cu and Co are finely tuned. The strongly coupled heterointerfaces (*i.e.*, Cu^0^/Cu^+^–CoO_*x*_ interfaces) and numerous active sites (such as Co^2+^–O_v_–Co^2+^, Cu^0^/Cu^+^, Ni–O–Co and/or Co_2_C) generated by this process may facilitate the formation of key *CO and *CH_*x*_ species, which are further converted into C_2+_ hydrocarbons. This study provides insights for optimizing Co-based catalysts for CO_2_ hydrogenation.

## Abstract 2

Ni modulates the phase composition of Cu–Co oxides, favoring active sites such as Cu^0^/Cu^+^ and Co^2+^–Ov–Co^2+^. This strengthens CO2 adsorption and the formation of *CO and *CH*x* intermediates, promoting the formation of C_2+_ hydrocarbons.

## Graphical abstract

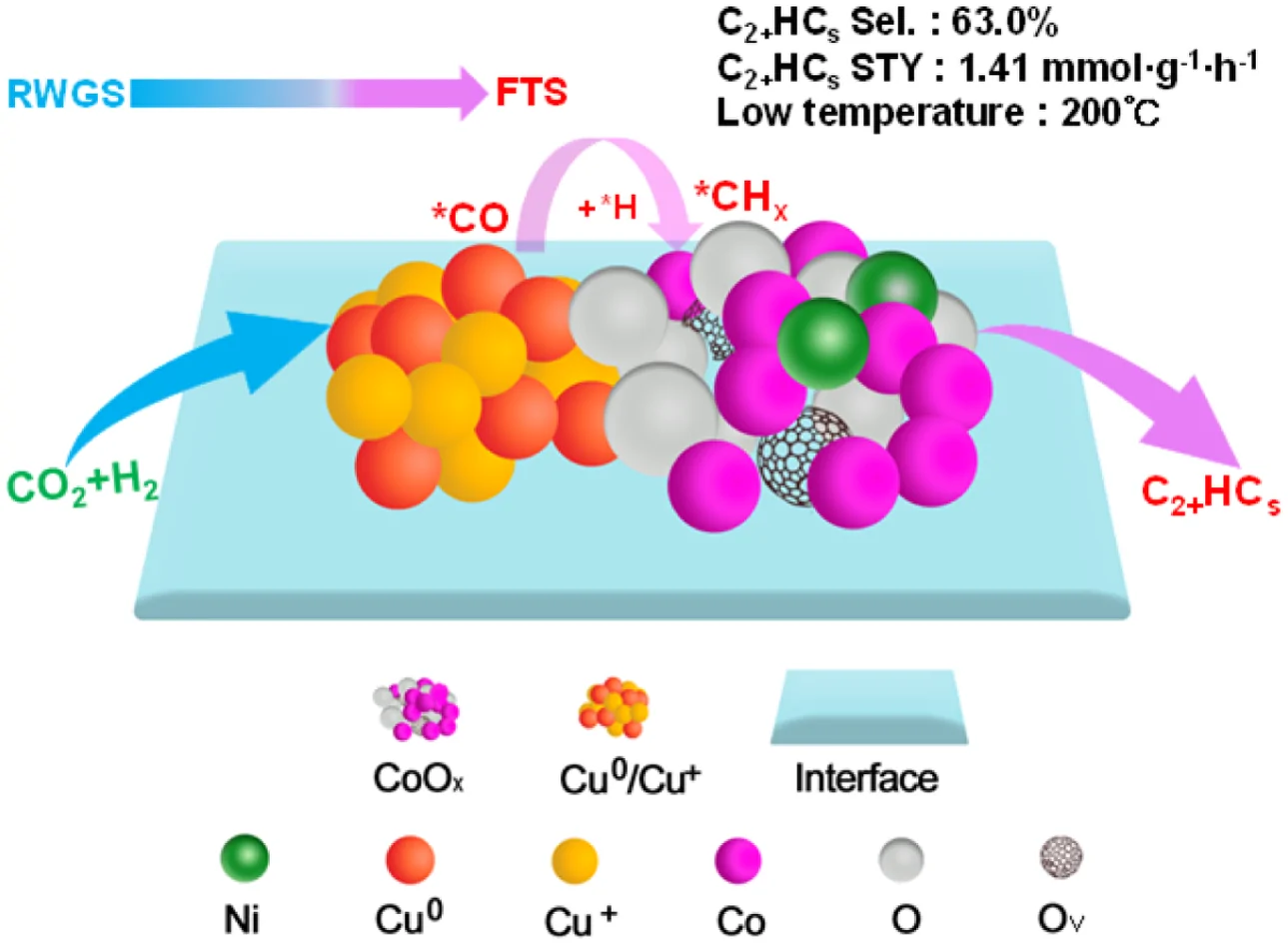

## 1 Introduction

CO_2_ is a well-known greenhouse gas and also an important renewable and readily available carbon resource. Conversion of CO_2_ to useful chemicals and fuels is a hot topic.^1^ Hydrogenation of CO_2_ to fuels and value-added chemicals may not only alleviate environmental issues associated with excessive CO_2_ emissions but also address fossil resource shortages.^2–4^ In most cases, CO_2_ is directly hydrogenated to C1 products, such as CO, methanol, methane, formic acid and its derivatives.^5–7^ Synthesis of products with two or more carbon atoms (C_2+_) from CO_2_ and H_2_ is a challenging task, because it involves controlled hydrogenation with simultaneous C–C bond formation.^8–13^ The C_2+_ hydrocarbons with high energy density exhibit broad application as fuels and chemicals, and the development of novel catalysts for CO_2_ hydrogenation to produce C_2+_ hydrocarbons represents a promising issue for sustainable development.

Currently, thermal catalytic hydrogenation of CO_2_ to C_2+_ hydrocarbons mainly involves two synthetic pathways.^3,14,15^ The first pathway is a modified Fischer–Tropsch route, where carbon dioxide undergoes the reverse water gas shift reaction (RWGS) to produce carbon monoxide, followed by the Fischer–Tropsch synthesis (FTS) to produce hydrocarbon compounds.^16–18^ Fe-based and Co-based catalysts are the main catalysts employed in the modified Fischer–Tropsch pathway.^19,20^ This route could be effectively improved by coupling the homogeneously catalyzed RWGS reaction and the heterogeneously catalyzed FTS reaction.^21^ The second route is through a methanol intermediate,^22^ where CO_2_ is first hydrogenated to methanol by metal oxide catalysts and subsequently converted into hydrocarbon products over molecular sieve-confined catalysis.^23–26^ It was also reported that CO_2_ can be directly hydrogenated to higher hydrocarbons *via* formation of CH_2_/CH_3_ monomers over the catalyst surface.^27^ Currently, developing catalysts capable of selectively producing C_2+_ hydrocarbons from CO_2_ hydrogenation at low temperatures is still highly desirable.^28^

Cobalt-based or Fe-based catalysts exhibit high efficiency in the Fischer–Tropsch synthesis (FTS) of C_5+_ alkanes from synthesis gas (CO/H_2_). However, during hydrogenation of CO_2_, the Fe or Co catalysts often demonstrate high methane selectivity.^29,30^ In the CO_2_-FTS pathway, transition metals such as Cu are usually introduced to enhance the performance of Fe-based catalysts in producing C_2+_ hydrocarbons.^31^ However, numerous studies on CO_2_/CO hydrogenation over Cu–Co catalysts predominantly resulted in production of alcohols.^32–37^ There are only limited reports of CuCo catalysts modified with alkali metals (Na or K) exhibiting improved performance for producing C_2+_ hydrocarbons at above 300 °C.^38,39^ Other new modification strategies for the CuCo catalysts are still needed.

As an earth-abundant metal, Ni catalysts are traditionally used to promote CO_2_ methanation.^40–42^ Yet, Ni doping can alter the surface structure of catalysts, generating new active sites or increasing the number of existing ones. For instance, in Ni–In catalysts, the intermetallic compound Ni_3_In formed between Ni and In possesses highly dispersed active sites, significantly enhancing methanol synthesis activity.^43^ In Ni–Fe bimetallic catalysts, the introduction of Ni promotes the conversion of CO_2_ to CH*, which is further hydrogenated to C_2_H_4_.^44^

In this work, we constructed a series of Ni-doped CuCo oxides as catalysts for the production of C_2+_ hydrocarbons *via* CO_2_ hydrogenation. Catalytic results indicate that the Ni_0.1_Cu_1_/CoO_*x*_ catalyst, synthesized with a Ni : Cu molar ratio of 0.1, exhibits a C_2+_ hydrocarbons selectivity of 63.0% at 200 °C, with a production rate of 1.41 mmol g_cat_^−1^ h^−1^. This performance ranks among the top level among the reported metal-based catalysts (Table S1). Combined characterization techniques revealed that Ni incorporation modulates the phase composition of the CuCo bimetallic catalyst, favoring the formation of Cu^0^/Cu^+^ and Co^2+^–O_v_–Co^2+^ (O_v_: oxygen vacancy) active sites on the catalyst surface. This significantly enhances CO_2_ surface adsorption and formation of *CO and *CH_*x*_ intermediate species, which are key for the FTS process to produce C_2+_ hydrocarbons. The modification of catalysts by introducing alkali metals mentioned above or transition metal promoters can change the surface properties of catalysts to generate abundant active sites. This strategy has some obvious advantages for the surface modification of catalysts.

## 2 Results and discussion

### 2.1 Catalyst characterization

A series of Ni_*n*_Cu_1_/CoO_*x*_ catalysts with varying Ni doping levels were synthesized *via* a combined simple coprecipitation and calcination method, where the molar ratio of Cu and Co was 1 : 6. The specific preparation process for the catalysts is shown in Fig. S1. The actual concentrations of Ni, Cu, and Co in the catalyst were determined using inductively coupled plasma-optical emission spectroscopy (ICP-OES). The actual mass ratios of Cu/Ni were close to the designed values (Table S2). Phase composition is a key factor influencing catalyst reactivity, and XRD analysis was applied to characterize the phase composition of the catalysts of different Ni contents, as well as the precipitate before and after calcination during preparation of the Ni_0.1_Cu_1_/CoO_*x*_ catalyst. As shown in Fig. 1a, the precipitate comprised the phases of Co_3_O_4_ (PDF#43-1003) and/or spinel NiCo_2_O_4_ (PDF#20-0781). Notably, NiCo_2_O_4_ with a spinel crystal structure could serve as an efficient catalyst. Due to the presence of Ni^2+^/Ni^3+^ and Co^2+^/Co^3+^ ions in multiple valence states on the surface, NiCo_2_O_4_ exhibited more pronounced redox properties than its constituent single-phase oxides (Co_3_O_4_ and NiO_2_).^45^

**Fig. 1:**
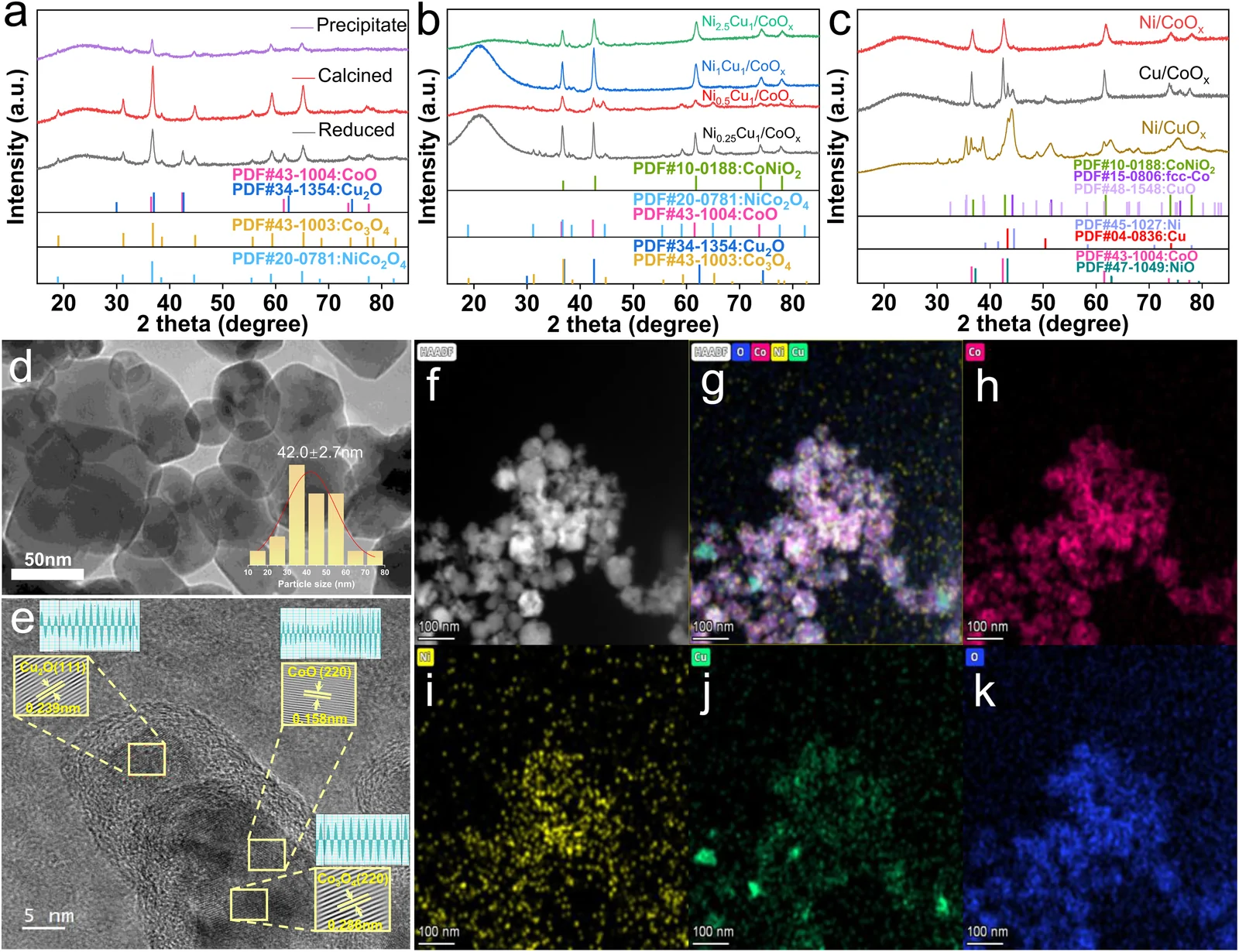
XRD patterns of (a) the precipitate, calcined precipitate, and the Ni_0.1_Cu_1_/CoO_*x*_ catalyst obtained after reduction, (b) the prepared Ni_0.25_Cu_1_/CoO_*x*_, Ni_0.5_Cu_1_/CoO_*x*_, Ni_1_Cu_1_/CoO_*x*_, and Ni_2.5_Cu_1_/CoO_*x*_ catalysts, and (c) the prepared Ni/CoO_*x*_, Cu/CoO_*x*_, and Ni/CuO_*x*_ catalysts. (d) TEM and particle size distribution of Ni_0.1_Cu_1_/CoO_*x*_. (e) High-Resolution (HR)TEM image of Ni_0.1_Cu_1_/CoO_*x*_, including its inverse fast Fourier transform (IFFT) images and lattice fringe distances (insets). (f–k) The STEM-HAADF and EDS mapping images of Co, Ni, Cu, and O in Ni_0.1_Cu_1_/CoO_*x*._

The calcined material simultaneously exhibited two identical phases. Following calcination, the characteristic diffraction peaks of Co_3_O_4_ slightly shifted toward higher angles. This shift occurred because moisture and organic matter were removed during calcination, leading to lattice contraction and consequently higher-angle peak shifts.^46*a*^ Additionally, the characteristic peak intensity of the precipitate was relatively weak, possibly due to poor crystallinity. The crystalline phases of materials after high-temperature calcination were more pronounced, and their crystallinity was improved. The reduced material exhibited four phases: Co_3_O_4_, CoO (PDF#43-1004), Cu_2_O (PDF#34-1354), and NiCo_2_O_4_. This suggests that the reduction process resulted in the formation of low-valent oxides of Co and Cu by reducing some Co_3_O_4_ and Cu species. In the reduced sample, the intensity of the characteristic peaks at 42.4°, 61.5°, and 73.7° corresponding to the (200), (220), and (311) planes of CoO obviously increased, while that of the peaks for Co_3_O_4_ decreased. The decrease in the oxidation state confirms the occurrence of the reduction process.

Fig. 1b displays the XRD patterns of samples with different Ni doping contents. At low doping levels, the Ni_0.1_Cu_1_/CoO_*x*_, Ni_0.25_Cu_1_/CoO_*x*_ and Ni_0.5_Cu_1_/CoO_*x*_ catalysts exhibited four distinct phases: Co_3_O_4_, CoO, Cu_2_O, and NiCo_2_O_4_. With the increase of the Ni doping levels, the peaks corresponding to Co_3_O_4_ gradually disappeared, and some NiCo_2_O_4_ phase may have transformed into CoNiO_2_ (PDF#10-0188). The XRD patterns of both Ni_0.25_Cu_1_/CoO_*x*_ and Ni_1_Cu_1_/CoO_*x*_ samples exhibit a broad hump at above 2*θ* ≈ 20°, which represent amorphous or long-range disordered materials originating from decreased crystallinity.^46*b*^ To compare with Ni_*n*_Cu_1_/CoO_*x*_ catalysts, Ni/CoO_*x*_, Cu/CoO_*x*_, and Ni/CuO_*x*_ were synthesized, and their XRD patterns are shown in Fig. 1c. Consistent with the literature, the Ni/CoO_*x*_ sample contained the CoNiO_2_ phase, while the Cu/CoO_*x*_ sample contained the fcc-Co^0^ (PDF #15-0806), Cu^0^ (PDF#04-0836) and CoO phases.^39^ Ni/CuO_*x*_ comprised three phases, including NiO (PDF#43-1004), Ni^0^ (PDF#45-1027), and CuO (PDF#48-1548). Notably, a comparison of Ni_0.1_Cu_1_/CoO_*x*_ and Cu/CoO_*x*_ samples revealed that Ni doping significantly influenced the phase composition of the material. The phases of the material transformed from fcc-Co^0^, Cu^0^, and CoO of the Cu/CoO_*x*_ sample to Co_3_O_4_, CoO, Cu_2_O, and NiCo_2_O_4_ of the Ni_0.1_Cu_1_/CoO_*x*_ sample. Consequently, the oxidation states of Co and Cu were also changed, which may affect catalytic performance.

Scanning electron microscopy (SEM) and transmission electron microscopy (TEM) techniques were employed to characterize the microstructure and particle size distribution of the catalysts, and the results are shown in Fig. S2–S4. The Ni_0.1_Cu_1_/CoO_*x*_ catalyst exhibited a nano-spherical particle morphology. At this precipitate stage, the particles exhibited a relatively loose aggregation state with indistinct boundaries between them, resulting in a larger specific surface area and high surface roughness. This structure may be attributed to the formation of disordered precipitates. The calcination process was typically conducted at high temperatures to remove organic impurities and moisture from the precipitate, thereby promoting particle crystallization and phase transformations. Thus the calcined Ni_0.1_Cu_1_/CoO_*x*_ nanoparticles had become more compact, the surface had grown smoother, and the boundaries between particles were more clear compared with precipitate before calcination, resulting in a more uniform nanoparticle structure.

The catalyst samples were tested by N_2_ adsorption (Fig. S5). The specific surface area and pore size of the packed catalysts are displayed in Table S2. After treatment of the calcined precipitate in a reducing atmosphere, the material's specific surface area increased and its pore size slightly decreased. Regarding the pore size distribution (Fig. S6), Ni_*n*_Cu_1_/CoO_*x*_ samples with varying Ni doping levels all exhibited broad pore size distributions. Their pore size range increased with increasing Ni doping. No micropores were detected in any sample, suggesting that the mesoporous channels likely originated from the stacking of catalyst particles.

The SEM and TEM images reveal the particle morphology of the prepared catalyst (Fig. S2 and S4). The reduction process may also change the internal structures and the oxidation states of the catalyst, which contribute to enhanced thermal catalytic activity.^47^ The prepared Ni_0.1_Cu_1_/CoO_*x*_ catalyst displayed an average particle size of 42.0 nanometers with a standard deviation of 2.7 nanometers (Fig. 1d). As the Ni doping concentration increased, remarkable agglomeration was observed, and the samples exhibited a relatively loose porous structure, such as Ni_0.5_Cu_1_/CoO_*x*_ and Ni_2.5_Cu_1_/CoO_*x*_ (Fig. S3). Without the Ni doping, the Cu/CoO_*x*_ samples showed severe agglomeration with scale-like morphology, which is similar to Ni/CoO_*x*_.

To gain a deeper understanding of the microstructure of Ni_0.1_Cu_1_/CoO_*x*_, HR-TEM, scanning transmission electron microscopy (STEM) and the corresponding EDS images were subsequently acquired. As observed in Fig. 1e, three distinct lattice spacings of 0.239 nm, 0.286 nm and 0.158 nm appear simultaneously, corresponding to the Cu_2_O(111), Co_3_O_4_(220) and CoO(220) crystal planes, respectively, which is consistent with the XRD results. Cu_2_O formed a close-packed heterointerface with cobalt oxides, creating a CuO_*x*_/CoO_*x*_ heterojunction structure. This structure indicates close contact between copper and cobalt oxide particles, triggering strong interactions between them. The possible presence of Cu–O–Co bond interfaces (as revealed by subsequent XAS analysis) provided electron transport pathways, facilitating charge transportation.^48^ The STEM-HAADF and EDS elemental mapping images revealed uniform spatial distributions of Co, Ni, Cu, and O elements (Fig. 1f–k). The HR-TEM images of Cu/CoO_*x*_ revealed three distinct lattice constants of 0.209 nm, 0.204 nm, and 0.213 nm corresponding to the Cu^0^(111), fcc-Co(111), and CoO(220) crystal planes, respectively (Fig. S7), which is consistent with XRD characterization. The doping of Ni markedly altered the phase structure of Cu/CoO_*x*._

The chemical state and local environment of Ni, Co and Cu in Ni_*n*_Cu_1_/CoO_*x*_ were further investigated by X-ray absorption near-edge structure (XANES) and extended X-ray absorption fine structure (EXAFS) spectroscopy.^49^ For Co K-edge XANES, the absorption edge of Ni_0.1_Cu_1_/CoO_*x*_ lies between those of CoO and Co_3_O_4_ (Fig. 2a). This indicates that the valence state of cobalt atoms lies between +2 and +3.^50^ The absorption edges of the three samples Ni_0.5_Cu_1_/CoO_*x*_, Ni_1_Cu_1_/CoO_*x*_ and Ni_2.5_Cu_1_/CoO_*x*_ are all located between those of CoO and Co-foil and are closer to those of CoO. These results are consistent with the XRD test results. The Fourier transform spectra from EXAFS (FT-EXAFS) of Ni_0.1_Cu_1_/CoO_*x*_ in Fig. 2d show that the first peak is located at around 1.50 Å, which can be attributed to Co–O interaction rather than Co–Co, as compared with those of Co_3_O_4_.^51^ The second shell peak (2.34 Å) of Co atoms in Ni_0.1_Cu_1_/CoO_*x*_ can be attributed to the Co–O–Co coordination structure, which is similar to those observed in Co_3_O_4_ and CoO. Notably, the second shell peak (2.95 Å) of Co atoms in the sample differs from that of the corresponding oxide reference samples, which may be due to the presence of a Co–O–Cu/Co coordination structure.^52^ The first shell peak (1.41 Å) and second shell peak (2.63 Å) of Ni_0.25_Cu_1_/CoO_*x*_ correspond to Co–O interaction and Co–O–Co coordination structures, respectively. The FT-EXAFS spectra of Ni_0.5_Cu_1_/CoO_*x*_, Ni_1_Cu_1_/CoO_*x*_ and Ni_2.5_Cu_1_/CoO_*x*_ samples are similar, which show weakened Co–O interaction. Moreover, a Co–Co coordination peak (2.17 Å) is observed in the samples with higher Ni contents, and the second shell peak is assigned to the Co–O–Co coordination structure. Thus, the Ni doping may remarkably alter the catalyst microstructure and valence of the Co.

**Fig. 2:**
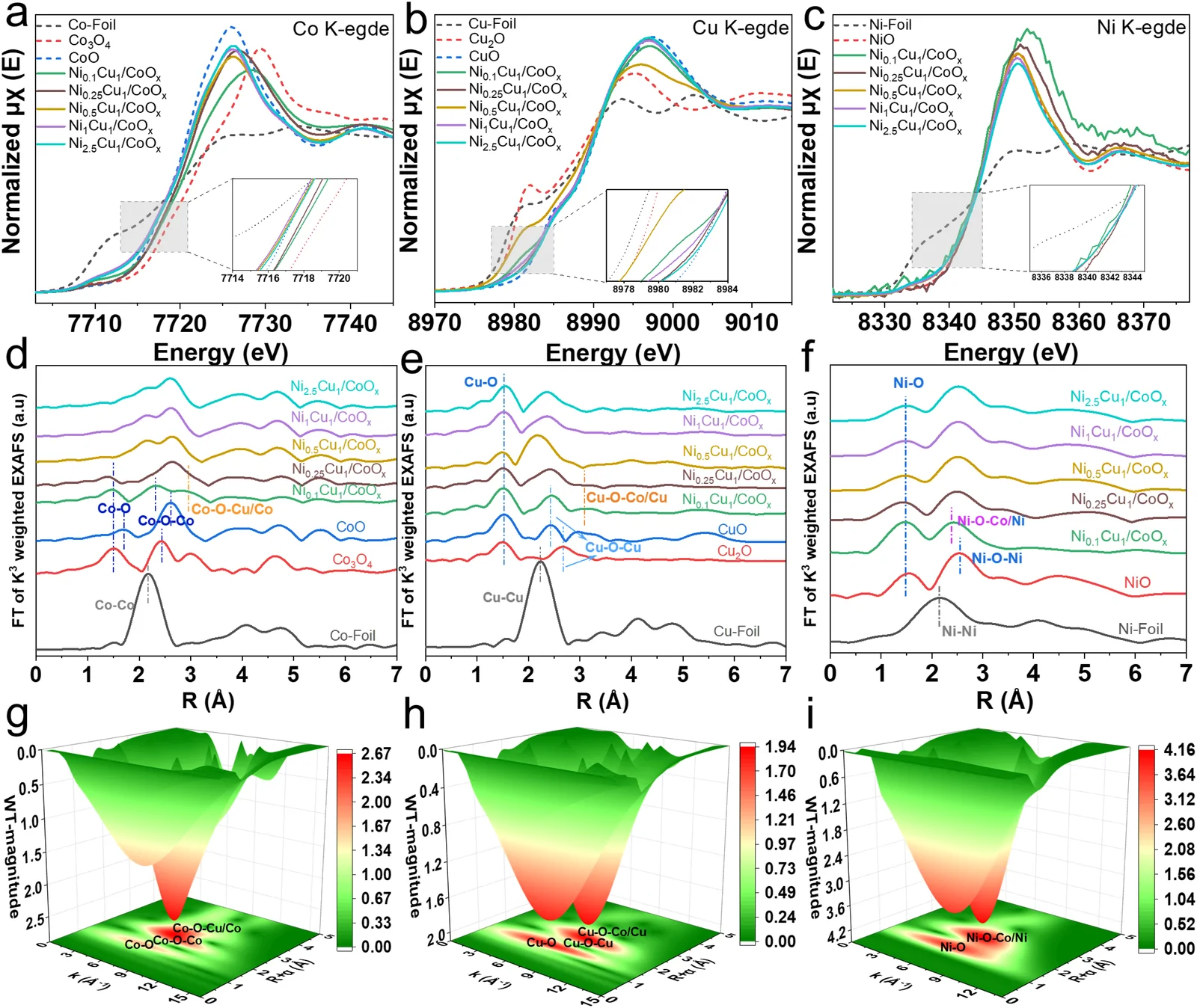
(a) Normalized Co K-edge XANES spectra (inset shows the magnified corresponding near-edge region), (b) normalized Cu K-edge XANES spectra (inset shows the magnified corresponding near-edge region), and (c) normalized Ni K-edge XANES spectra (inset shows the magnified corresponding near-edge region) of the Ni_*n*_Cu_1_/CoO_*x*_ catalysts. (d) FT magnitude *k*^3^-weighted Co K-edge EXAFS spectra, (e) FT magnitude *k*^3^-weighted Cu K-edge EXAFS spectra, and (f) FT magnitude *k*^3^-weighted Ni K-edge EXAFS spectra of the Ni_*n*_Cu_1_/CoO_*x*_ catalyst. (g–i) WT-EXAFS spectra of Co K-edge, Cu K-edge, and Ni K-edge for the Ni_0.1_Cu_1_/CoO_*x*_ catalyst, respectively.

Similarly, by comparing the Cu K-edge XANES spectra, the average oxidation state of the Cu compound in all Ni_0.1_Cu_1_/CoO_*x*_ samples lies between +1 and +2 (Fig. 2b). A dominant peak at 1.52 Å corresponding to the Cu–O bond is detected in all Ni_*n*_Cu_1_/CoO_*x*_ samples (Fig. 2e). For the Ni_0.1_Cu_1_/CoO_*x*_ sample, the main peak from the second coordination layer is located at 2.48 Å, and a secondary peak is located at 3.10 Å. This indicates that the Cu–O–Cu and Cu–O–Co coordination structures may coexist in the Ni_0.1_Cu_1_/CoO_*x*_ sample.^52,53^ As Ni doping increased, the Cu–O–Co/Cu coordination peak in Ni_*n*_Cu_1_/CoO_*x*_ samples became weak and disappeared finally.

Additionally, the Ni K-edge peak in the XANES spectrum (Fig. 2c) indicates that the valence state of Ni atoms in all Ni_*n*_Cu_1_/CoO_*x*_ samples is very close to the +2. A dominant peak at 1.48 Å corresponding to the Ni–O bond is detected in all Ni_*n*_Cu_1_/CoO_*x*_ samples (Fig. 2f), which is close to the signal peak (1.53 Å) of the reference sample NiO. For the Ni_0.1_Cu_1_/CoO_*x*_ sample, the main peak from the second coordination layer is located at 2.41 Å, differing from the Ni–O–Ni coordination structure (2.55 Å) in NiO, suggesting the possible existence of a Ni–O–Co/Ni coordination structure.^54–56^ The second shell of the remaining samples belongs to the Ni–O–Ni structure. Low-concentration Ni doping promotes the formation of a Ni–O–Co bond, facilitating electron transport and regulating the electronic structure of the Cu–Co oxide system. In contrast, Ni/CoO_*x*_ alone forms Ni–O and Ni–O–Ni coordination structures, similar to the coordination environment of the reference NiO sample (Fig. S8).

Furthermore, the wavelet transform was employed to further illuminate the Co, Cu and Ni K-edges EXAFS oscillation of the Ni_0.1_Cu_1_/CoO_*x*_ samples in both *R* and *K* space resolution. In general, the positions (*i.e.*, *R* and *K* values) of the maximum intensity in WT-EXAFS images are chiefly correlated with the bond length and atomic number of scattering atoms.^57^ The wavelet transform of the *k*^3^-weighted EXAFS signals for Co K-edge, Cu K-edge, and Ni K-edge spectra (Fig. 2g–i) also clearly reveals the formation of Co–O–Cu/Co, Cu–O–Co/Cu, and Ni–O–Co/Ni bonds in the Ni_0.1_Cu_1_/CoO_*x*_ sample.

To further confirm the modulation of material phases by Ni doping and its subsequent impact on the coordination environment, the Cu/CoO_*x*_ sample was also tested (Fig. 3). The Co K-edge peak in the XANES spectrum (Fig. 3a) shows that the absorption edge position of Cu/CoO_*x*_ lies between that of Co foil and CoO. This indicates that the valence state of cobalt atoms is between 0 and +2. This is consistent with XRD findings, confirming that the Cu/CoO_*x*_ sample contains both fcc-Co and CoO phases. The FT-EXAFS spectrum (Fig. 3b) shows a first peak at ≈1.51 Å with relatively weak intensity, attributed to Co–O interactions.^51,58^ The second shell main peak (2.16 Å) of Co atoms in Cu/CoO_*x*_ can be attributed to the Co–Co coordination structure. The second shell peak (2.78 Å) of Co atoms can be attributed to the Co–O–Co coordination structure. The wavelet transform of the *k*^3^-weighted EXAFS signal from the Co K-edge spectrum (Fig. 3c) clearly reveals the formation of a Co–Co bond in the Cu/CoO_*x*_ sample. Similarly, by comparing the Cu K-edge XANES spectra, the oxidation state of Cu in the Cu/CoO_*x*_ samples ranges between +1 and +2, with a preference for +1 (Fig. 3d).^59^ The FT-EXAFS spectrum of the Cu/CoO_*x*_ sample exhibits a peak at approximately 1.51 Å (Fig. 3e), which can be attributed to Cu–O interaction. Additionally, a major peak appears at 2.18 Å, closely corresponding to the Cu–Cu bond length and similar to the peak observed in the reference sample Cu-foil (2.21 Å). Furthermore, the formation of the Cu–Cu bond in the Cu/CoO_*x*_ sample was also observed in wavelet transform of the *k*^3^-weighted EXAFS signal for the Cu K-edge spectrum (Fig. 3f).

**Fig. 3:**
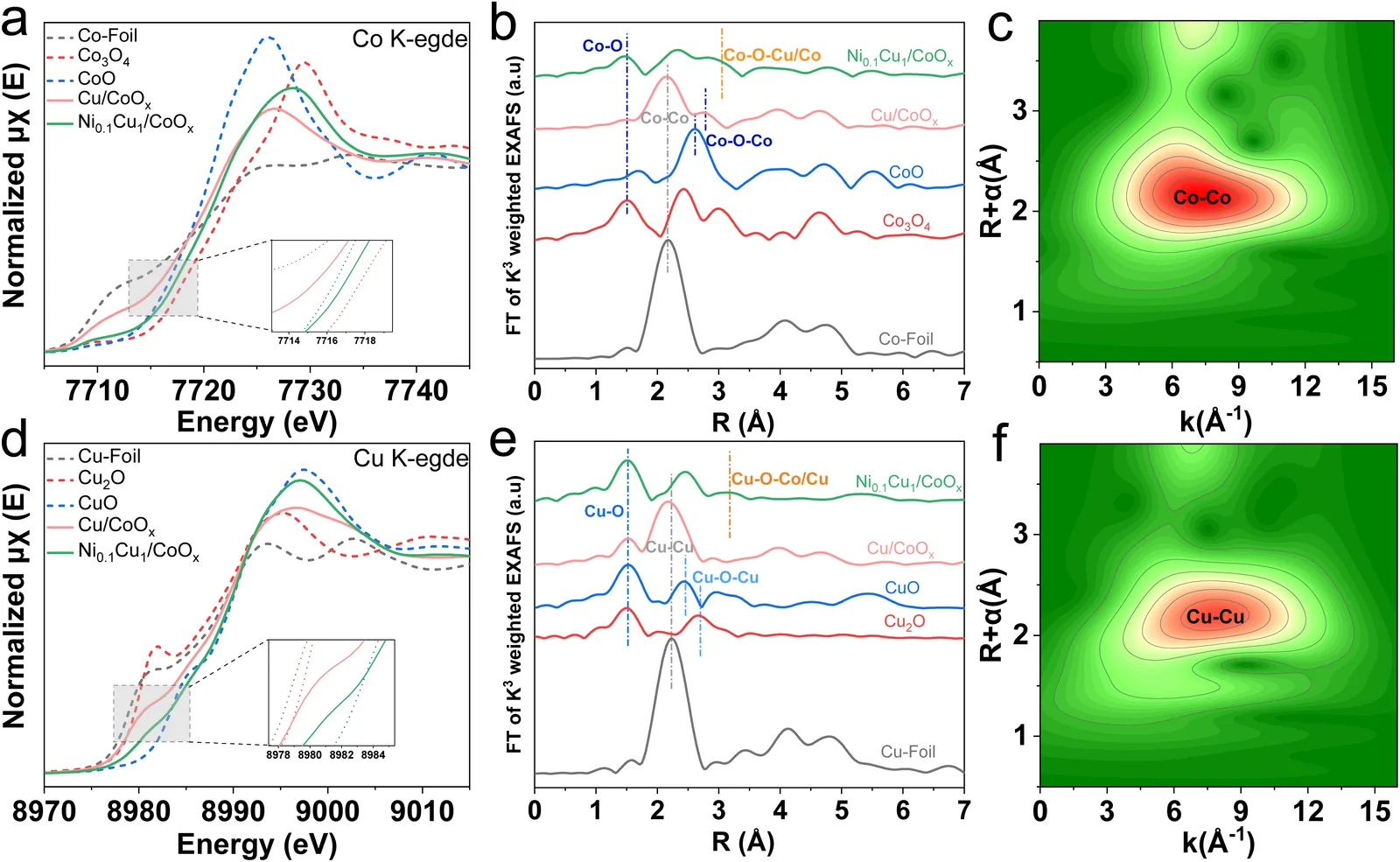
(a) Normalized Co K-edge XANES spectra (inset shows the magnified corresponding near-edge region) and (b) Fourier transform (FT) magnitude *k*^3^-weighted Co K-edge EXAFS spectra of the Ni_0.1_Cu_1_/CoO_*x*_ and Cu/CoO_*x*_ catalysts. (c) WT-EXAFS spectra of Co K-edge for Cu/CoO_*x*_. (d) Normalized Cu K-edge XANES spectra (inset shows the magnified corresponding near-edge region) and (e) FT magnitude *k*^3^-weighted Cu K-edge EXAFS spectra of the Ni_0.1_Cu_1_/CoO_*x*_ and Cu/CoO_*x*_ catalysts. (f) WT-EXAFS spectra of Cu K-edge for Cu/CoO_*x*_.

The combined spectral results indicate that, unlike Cu/CoO_*x*_, the Ni_0.1_Cu_1_/CoO_*x*_ sample with lower Ni doping level contains NiCo_2_O_4_ spinel and Co_3_O_4_ phases, which are more conducive to forming CuO_*x*_/CoO_*x*_ heterojunction structures. This alteration in the coordination environment facilitates the formation of Cu–O–Co and Ni–O–Co coordination structures, promoting electron transport and thereby contributing to the generation of surface active sites. The reduction step of the calcined Ni_0.1_Cu_1_/CoO_*x*_ sample is also crucial to the formation of the surface active sites (Fig. S9 and S10).

X-ray photoelectron spectroscopy (XPS) characterization was employed to further investigate the elemental composition and electronic structure of catalysts. The high-resolution Co XPS spectra of Ni_0.1_Cu_1_/CoO_*x*_ (Fig. 4a) present three pairs of peaks, corresponding to Co^3+^ 2p_3/2_ and Co^3+^ 2p_1/2_ (779.3 and 794.2 eV), Co^2+^ 2p_3/2_ and Co^2+^ 2p_1/2_ (780.7 and 795.7 eV), and satellite signals (787.3 and 803.5 eV). As nickel doping increases, the cobalt peak shifts toward higher binding energies, indicating electron transfer from Co to Ni at higher Ni doping levels. Electron transfer from Co to Ni occurs *via* the Co–O–Ni oxygen bridge structure. However, at low Ni doping level of Ni_0.1_Cu_1_/CoO_*x*_, the electrons transfer from Ni to Co, leading to increased formation of the Co^2+^-O_v_-Co^2+^ structure (O_v_: oxygen vacancy).^60–62^ Because the Co^2+^/Co^3+^ ratio of the Cu/CoO_*x*_ sample is 1.22 (Fig. S11), which is enhanced to 1.33 with the introduction of a small amount of Ni in Ni_0.1_Cu_1_/CoO_*x*_ (Fig. 4a). The Cu XPS spectra (Fig. 4b) present three pairs of peaks, corresponding to Cu^+^ 2p_3/2_ and Cu^+^ 2p_1/2_ (932.2 and 952.0 eV), Cu^2+^ 2p_3/2_ and Cu^2+^ 2p_1/2_ (934.1 and 954.0 eV), and satellite signals (941.8 and 961.5 eV), respectively. Similarly, as the Ni content increases, the copper peak shifts toward higher binding energies. This indicates electron transfer from the CuO_*x*_ surface to Ni species. Conversely, at low Ni contents of Ni_0.1_Cu_1_/CoO_*x*_, electrons transfer from Ni species to CuO_*x*_*via* the Co–O–Cu bond, creating a more negative Cu surface with a higher Cu^0^/Cu^+^ ratio (Fig. 4c and S11c).

**Fig. 4:**
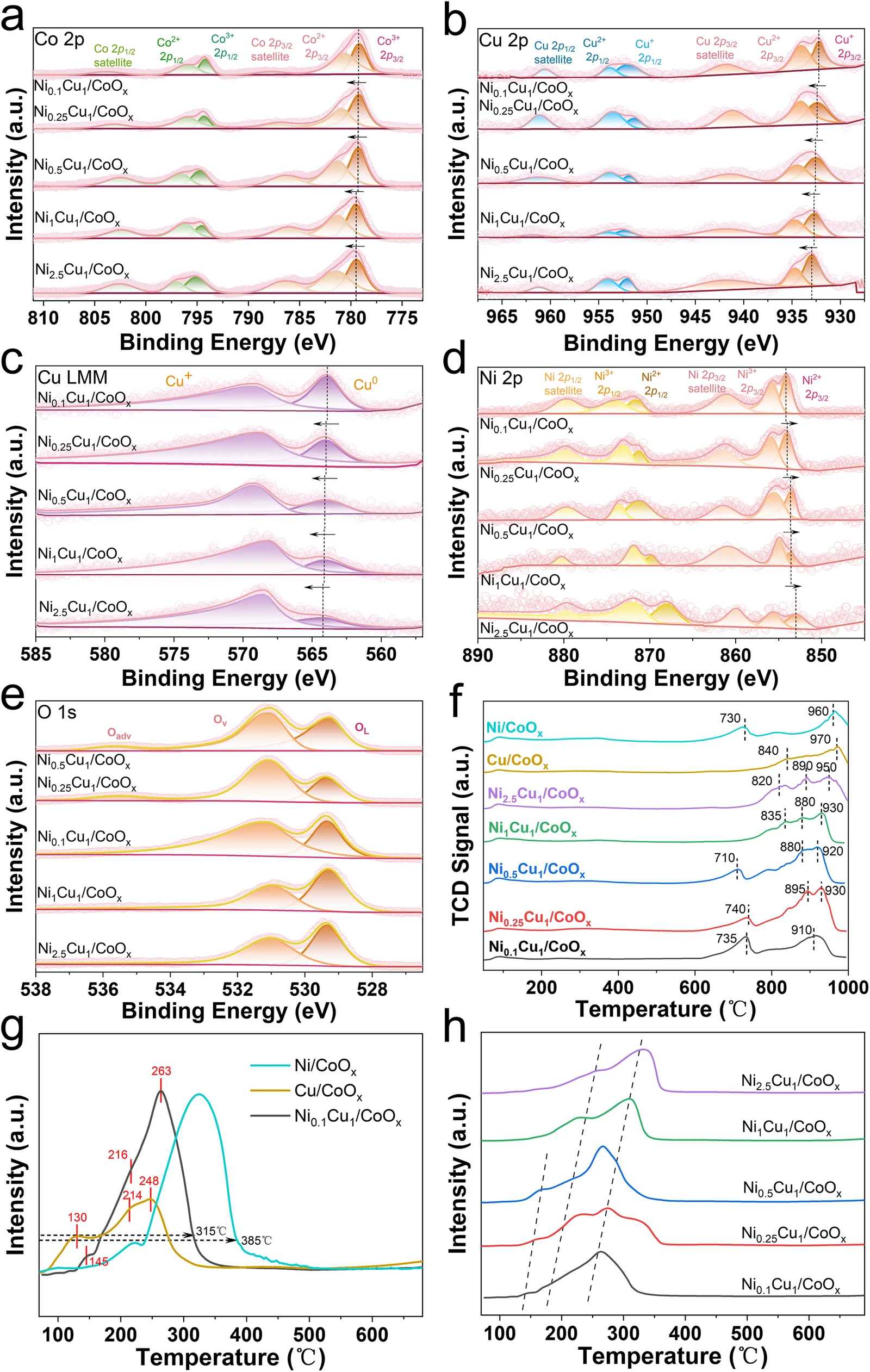
(a) Co 2p XPS spectra of the Ni_*n*_Cu_1_/CoO_*x*_ catalysts. (b) Cu 2p XPS spectra of the Ni_*n*_Cu_1_/CoO_*x*_ catalysts. (c) Cu LMM Auger spectra of the Ni_*n*_Cu_1_/CoO_*x*_ catalysts. (d) Ni 2p XPS spectra of the Ni_*n*_Cu_1_/CoO_*x*_ catalysts. (e) O 1s XPS spectra of the Ni_*n*_Cu_1_/CoO_*x*_ catalysts. (f) The CO_2_-TPD profiles of the Ni/CoO_*x*_, Cu/CoO_*x*_, and Ni_*n*_Cu_1_/CoO_*x*_ catalysts. (g) The H_2_-TPR profiles of the Ni/CoO_*x*_, Cu/CoO_*x*_, and Ni_0.1_Cu_1_/CoO_*x*_ catalysts. (h) The H_2_-TPR profiles of the Ni_*n*_Cu_1_/CoO_*x*_ catalysts.

The Ni XPS spectra (Fig. 4d) reveal that the nickel content increase shifts the Ni 2p peak toward lower binding energies, confirming the effect of nickel species on capturing electrons from cobalt and copper at higher Ni doping levels. As depicted in Fig. 4e, the fine O 1s spectra of Ni_0.5_Cu_1_/CoO_*x*_ samples show three deconvoluted peaks at about 529.3, 531.1, and 535.4 eV, which are ascribed to lattice oxygen species (O_L_), surface oxygen defects or oxygen vacancies (O_v_), and adsorbed surface hydroxyl groups (O_adv_), respectively.^63^ Noticeably, as the Ni doping concentration decreases, the increase in the O_v_/O_L_ intensity indicates the generation of more oxygen vacancies. Compared to Cu/CoO_*x*_ samples, doping with low Ni content promotes the formation of oxygen vacancies, which may act as active sites of the catalyst. Collectively, these phenomena indicate strong electron interactions between Ni and the CuO_*x*_/CoO_*x*_ heterojunction, which may remarkably affect the catalytic performance.^63^

H_2_-TPR was employed to characterize the redox properties of the samples. Fig. 4g shows three reduction peaks in the Cu/CoO_*x*_ sample. The hydrogen consumption peak at 130 °C corresponds to the Cu^2+^ → Cu^+^ reduction, the peak at 214 °C corresponds to the Cu^+^ → Cu^0^ reduction, and the peak at 248 °C corresponds to the Co^3+^ → Co^2+^ transformation. In the Ni-doped Ni_0.1_Cu_1_/CoO_*x*_ material, all three reduction peaks are shifted toward higher temperatures, though the shift is modest. This indicates that Ni exerts a certain influence on the reduction process in the Cu–Co oxide system. It slightly weakens the Cu^2+^ → Cu^+^ reduction while enhancing the Cu^+^ → Cu^0^ reduction, thereby favoring the formation of Cu^0^/Cu^+^ active sites.^39^ There is a significant increase in the reduction peak of Co oxides, indicating strong interactions between Ni and Co species. This may be attributed to the formation of a NiCo_2_O_4_ spinel structure that promotes the reduction degree of Co species.^62^ Overall, Ni not only acts as a structural promoter to enhance Cu oxide reduction but also weakens Co–O bonds to improve Co_3_O_4_ reduction. The samples with varying Ni doping levels were also tested (Fig. 4h), and all reduction peaks shifted toward higher temperatures as Ni doping increased, which is consistent with the H_2_-TPR result of Ni/CoO_*x*_. This is likely due to the formation of a new phase, CoNiO_2_, at a higher Ni doping level.^64^

### 2.2 Catalytic performance

The catalysts were appraised in batch reactors. Fig. 5a shows the effects of different catalytic components on the CO_2_ hydrogenation reaction, aiming to investigate the roles of the metals in the catalyst. The experimental data indicate that catalysts containing only single metallic Ni or Co oxides produce very little C_2+_ hydrocarbons. The NiO_*x*_ and Ni/CuO_*x*_ catalysts tended to generate CO, with higher selectivity for CH_4_ in hydrocarbon products. Compared with Ni and Cu, the metal CoO_*x*_ catalyst possessed lower CO selectivity, and a slightly higher formation rate of C_2+_ hydrocarbons with a remarkably higher C_2+_ selectivity in hydrocarbon distribution. The tendency of the CoO_*x*_ catalyst to generate C_2+_ hydrocarbons could be greatly enhanced by combining with Cu in the Cu/CoO_*x*_ catalyst, and this phenomenon has been reported elsewhere.^38,39^ After testing the catalytic performances of the Cu/CoO_*x*_ catalysts with different Cu/Co ratios, it was confirmed that the Cu/Co ratio of 1 : 6 was appropriate (Fig. S12). In contrast, a combination of Ni and Co in the Ni/CoO_*x*_ catalyst was not as effective as the combination of Cu and Co in the Cu/CoO_*x*_ catalyst. Nevertheless, introduction of a small amount of Ni to Cu/CoO_*x*_ to form the Ni_0.1_Cu_1_/CoO_*x*_ catalyst (NiCuCo) could further markedly elevate the catalytic performance. The selectivity of C_2+_ hydrocarbon products reaches 63.0%, with a C_2+_ activity of 1.41 mmol g_cat_^−1^ h^−1^ and CO_2_ conversion of 17.5%. As we have discussed above, at a low Ni doping level of Ni_0.1_Cu_1_/CoO_*x*_, the electrons transfer from Ni to Co, leading to increased formation of the Co^2+^–O_v_–Co^2+^ structure (O_v_: oxygen vacancy); moreover, electrons transfer from Ni species to CuO_*x*_*via* the Co–O–Cu bond, creating a more negative Cu surface with a higher Cu^0^/Cu^+^ ratio. These structures may act as active sites for the formation of C_2+_ hydrocarbons, accounting for the promoting effect of Ni doping. The molar ratio of Ni/Cu in the Ni_*n*_Cu_1_/CoO_*x*_ catalyst could evidently affect the catalytic performance (Fig. 5b). As the Ni doping amount increased, the selectivity and activity of C_2+_ hydrocarbon products of the catalysts exhibited two different volcano-shaped trends, respectively. At a Ni/Cu ratio of 0.5 : 1, the formation rate of C_2+_ hydrocarbon products reached its highest value. While for the selectivity of C_2+_ hydrocarbons, the highest value of 63.0% was obtained at a Ni/Cu ratio of 0.1 : 1. The complicated behavior of the catalysts may be mainly attributed to two structural aspects, *i.e.*, the specific surface area and the chemical structure of the catalyst surface. Compared to Cu/CoO_*x*_ (13.7 m^2^ g^−1^), the specific surface area of Ni_0.1_Cu_1_/CoO_*x*_ increased to 26.9 m^2^ g^−1^. Although Ni doping evidently enhanced the activity per unit mass, the surface-area-normalized activity actually decreased (Table S3). One possible reason is that a higher specific surface area is accompanied by smaller pores, which slows down mass transport and reduces the utilization of the surface. As we have discussed in the former paragraphs, the Ni doping also markedly modified the nature of the catalyst surfaces. The overall activity and selectivity of the target products are jointly determined by the physical and chemical properties of the catalyst.

**Fig. 5:**
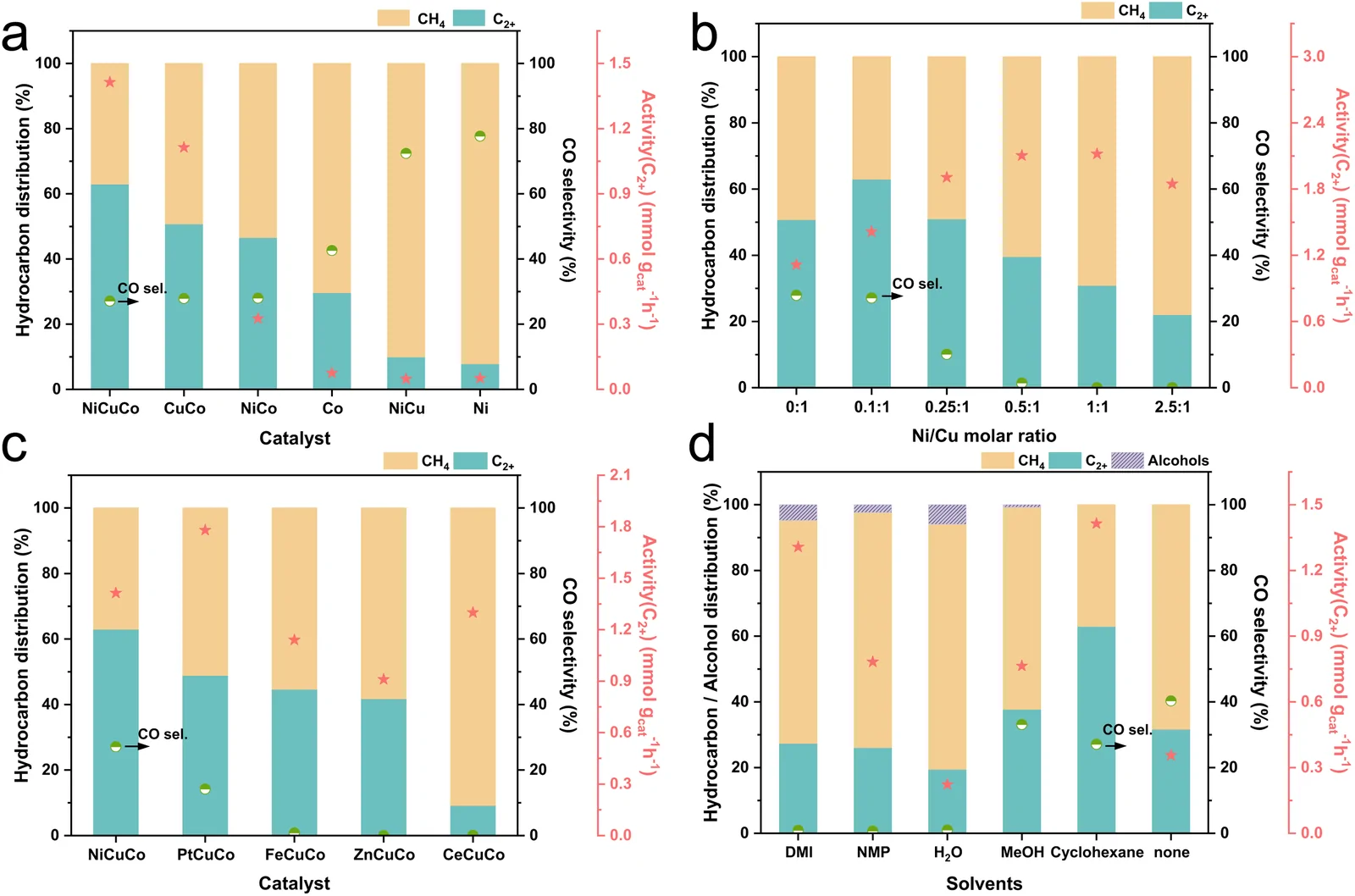
(a) Effects of different metal combinations, (b) different Ni doping levels, (c) different doping metals, and (d) different solvents on CO_2_ hydrogenation. Reaction conditions: 50 mg catalyst and 2 mL of cyclohexane (unless otherwise stated), an initial pressure of 8.0 MPa (CO_2_/H_2_ = 1 : 1), 12 h, and 200 °C. (Note: all metal combinations in the figure are the corresponding oxide catalysts. The trimetallic MCuCo catalyst indicates M_0.1_Cu_1_/CoO_*x*_, where M represents Ni, Pt, Fe, Zn and Ce. The ratios of Ni/Co, Ni/Cu and Cu/Co in the bimetallic catalysts were 1/6).

We examined the catalysts using CO_2_-TPD, as shown in Fig. 4f; in all catalysts, adsorption peaks at higher temperature regions above 460 °C were found, indicating a strong surface interaction between the basic site of the catalyst and acidic CO_2_.^65,66^ By comparing Cu/CoO_*x*_ and Ni_0.1_Cu_1_/CoO_*x*_ samples, it was found that Ni doping enhances CO_2_ adsorption on the catalyst surface. This adsorption may be facilitated by the formation of surface structures such as Co^2+^–O_v_–Co^2+^ and Ni–O–Co. However, excessive Ni doping hinders adsorption and dissociation of the H_2_ molecule, potentially reducing catalyst activity (Fig. 4h). H_2_ activation is a critical step in the hydrogenation of carbon-containing species, thus affecting the CO_2_ conversion and product selectivity.

Fig. 5c shows the effect of different doping metals on the performance of Cu/Co oxide catalysts in CO_2_ hydrogenation. In contrast to Ni_0.1_Cu_1_/CoO_*x*_, doping with Pt enhanced the activity of C_2+_ hydrocarbons to some extent, but the selectivity of C_2+_ products decreased obviously. When other metals (Fe, Zn, Ce, *etc*.) were doped, both the activity and selectivity of C_2+_ hydrocarbons were lower than those of Ni_0.1_Cu_1_/CoO_*x*_. Overall, the Ni_0.1_Cu_1_/CoO_*x*_ catalyst exhibited the optimal catalytic performance in this reaction. Moreover, the catalyst was appraised in the liquid solid phase reaction of CO_2_ and H_2_, where the solvent played an important role (Fig. 5d). The solvent may not only affect the mass and heat transfer but also influence the activity and selectivity of the reaction. H_2_O as the reaction solvent may inhibit the selectivity and activity for C_2+_ hydrocarbon products. In contrast, methanol as the solvent could gently increase both activity and selectivity of the desired products. Other solvents such as DMI and NMP may promote the formation rates of C_2+_ hydrocarbon, but the related selectivities dropped slightly. The non-polar solvent cyclohexane demonstrated the best selectivity and activity of C_2+_ hydrocarbon products among all the tested solvents. This may be ascribed to the principle of like dissolves like. Using cyclohexane as the reaction solvent is more conducive to the formation of C_2+_ hydrocarbon products and desorption of hydrocarbons from the catalyst surface. The solvent may also affect the sorts of product in the reaction.^27^ Alcohol byproducts such as methanol and ethanol could be detected after the reaction in polar solvents such as DMI, NMP, H_2_O and methanol.

Based on the optimal catalyst and solvent, we studied other reaction conditions such as reaction temperature, gas pressure and reaction time. Fig. 6a shows the effect of reaction temperature on reaction activity and selectivity. Compared with the higher temperatures required for reported CO_2_ hydrogenation reactions to produce C_2+_ long-chain hydrocarbons, our catalytic system can promote the reaction at a lower temperature of around 180 °C. The reaction can proceed within the temperature range of 180–220 °C, with the selectivity of C_2+_ hydrocarbons exhibiting a volcanic-type trend. At 200 °C, the selectivity of C_2+_ hydrocarbon products reaches its peak at 63.0%. Therefore, 200 °C is the optimal reaction temperature. As shown in Fig. 6b, the effect of reaction time on the catalytic reaction was investigated. The study showed that from 3 to 12 h, the selectivity of CO gradually decreased, while the selectivity and formation rate of C_2+_ hydrocarbon products gradually increased. After 12 h, the production rate and selectivity of C_2+_ hydrocarbons started to decrease. Therefore, a reaction time of 12 h was appropriate. We further investigated the effect of total pressure when CO_2_ and H_2_ were in equal proportions (Fig. 6c). As the total pressure increased, the catalyst activity kept growing. At 8 MPa, the activity reached its peak, and the selectivity of C_2+_ hydrocarbon products was also the highest at this point. We further fixed the total pressure at 8 MPa and studied the effect of the CO_2_/H_2_ ratio on the reaction (Fig. 6d). As the CO_2_ pressure increased, the gas ratio changed from 2/6 to 4/4, and the activity and selectivity of C_2+_ hydrocarbon products showed an upward trend, reaching a peak at a ratio of 4/4. When the CO_2_/H_2_ ratio exceeded 4/4, the decrease of the desired catalytic results began, although the selectivity of the byproduct CO continued to rise. Therefore, a CO_2_/H_2_ ratio of 4 MPa/4 MPa was optimal for the reaction.

**Fig. 6:**
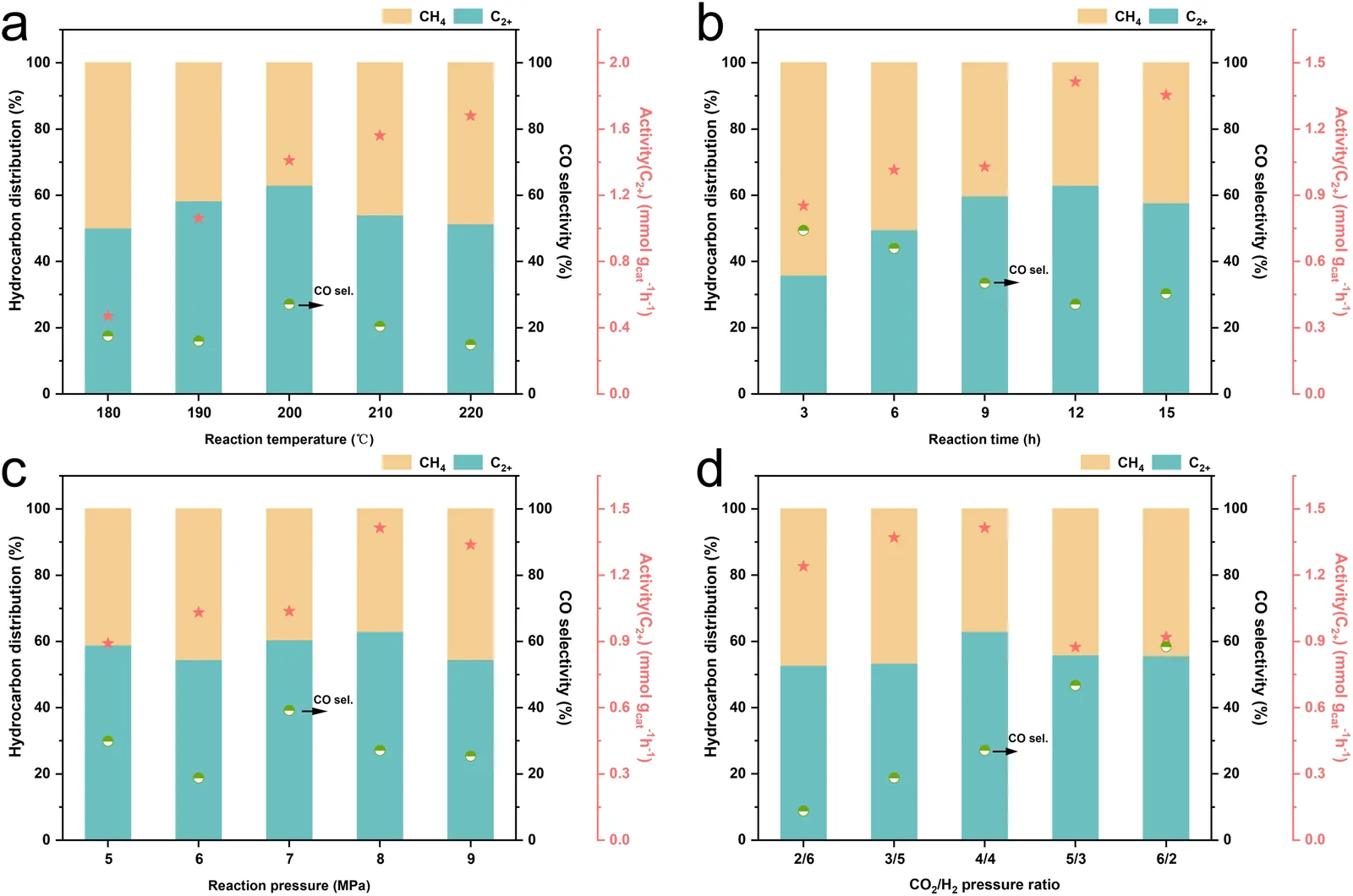
Effect of reaction conditions [(a) reaction temperature, (b) reaction time, (c) total pressure, and (d) CO_2_/H_2_ ratio] on CO_2_ hydrogenation using 50 mg of Ni_0.1_Cu_1_/CoO_*x*_ catalyst and 2 mL of cyclohexane. Other conditions: (a) 4 MPa CO_2_ and 4 MPa H_2_, 12 h; (b) 4 MPa CO_2_ and 4 MPa H_2_, 200 °C; (c) *P*_CO_2__/*P*_H_2__ = 1, 200 °C, 12 h; (d) total pressure of 8 MPa, 200 °C, 12 h.

Under the optimal conditions, the selectivity of C_2+_ hydrocarbon products was as high as 63.0%, with a C_2+_ activity of 1.41 mmol g_cat_^−1^ h^−1^ and CO_2_ conversion of 17.5%. The GC analysis results of gas and liquid products are shown in Fig. S13–S15. The C_2+_ product distribution and the Anderson–Schulz–Flory (ASF) plot of the Ni_0.1_Cu_1_/CoO_*x*_ catalyst in CO_2_ hydrogenation under optimal conditions exhibited a chain growth probability (*α*) of 0.47 and a coefficient of determination *R*^2^ = 0.97 (Fig. 7a). Interestingly, the final C_2+_ products in this work were entirely straight-chain hydrocarbons including alkanes and alkenes, with a preference for straight-chain alkanes. And the largest straight-chain alkane observed was *n*-heptadecane. In previously reported CO_2_ hydrogenation for synthesis of liquid fuels, the products typically contained various compounds, such as alkenes, aromatics, CO, straight-chain alkanes, branched-chain alkanes, cycloalkanes and oxygen-containing compounds. The product distribution of this work differed from that of similar metal oxide catalysts.^16,23,27,67^ We would like to mention that the presence of solvent in this work may affect the sort of products, because when we conducted this reaction in a fixed-bed reactor without a solvent, minor oxygen-containing products such as methanol and ethanol were observed (Fig. S16). This indicated that using cyclohexane as the reaction solvent prevented the generation of the oxygen-containing byproducts. Finally, we investigated the recycling stability of the Ni_0.1_Cu_1_/CoO_*x*_ catalyst, and the results are shown in Fig. 7b. The selectivity of C_2+_ hydrocarbons maintained at about 60%, and the activity did not decline obviously after three runs, which suggested the good stability of the catalyst.

**Fig. 7:**
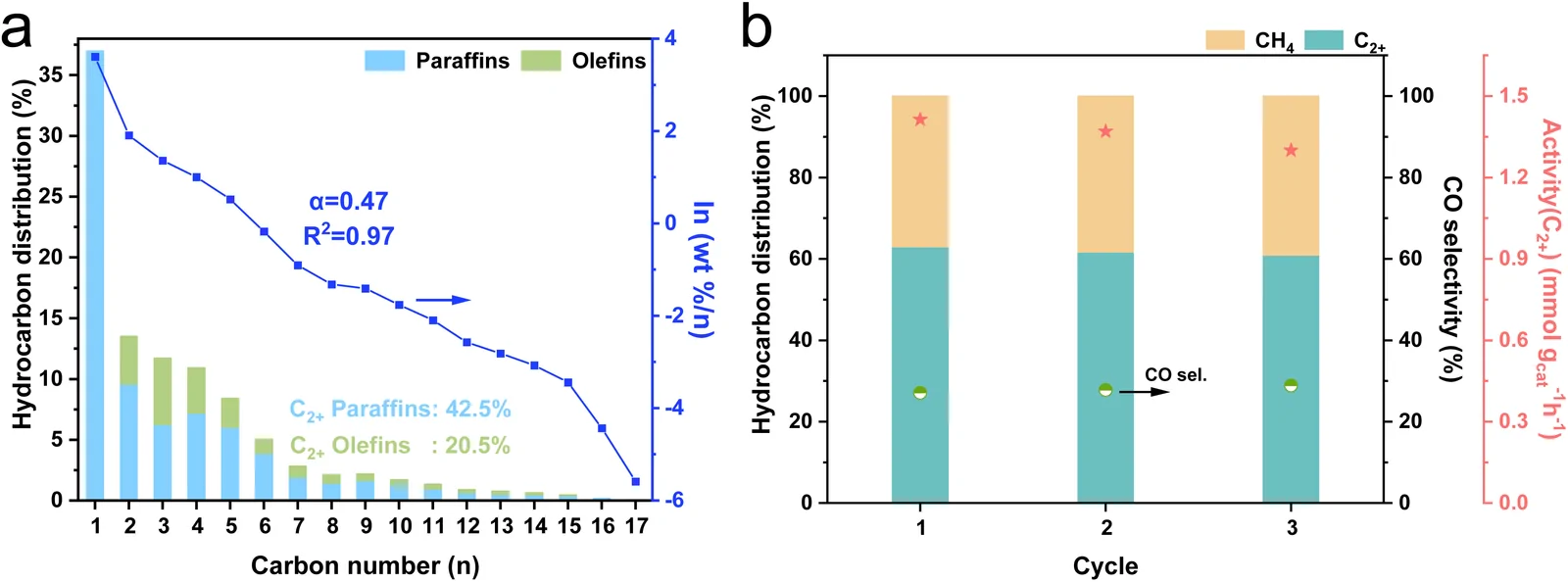
(a) The detailed hydrocarbon distribution and the Anderson–Schulz–Flory plot of the reaction product over the Ni_0.1_Cu_1_/CoO_*x*_ catalyst under the optimal reaction conditions. The hydrocarbons consisted of *n*-paraffins and minor linear monoolefins. The selectivity of C_2+_ paraffins in the C_2+_ products was 42.5%, and the selectivity of olefins in the C_2+_ products was 20.5%. (b) The results of the recycling experiments with the Ni_0.1_Cu_1_/CoO_*x*_ catalyst; reaction conditions: 50 mg of catalyst, 2 mL of cyclohexane, initial pressure of 8.0 MPa (CO_2_/H_2_ = 1 : 1), 12 h, and 200 °C.

### 2.3 Mechanistic discussion

To investigate the metal valence state and surface composition of the catalyst under the reaction conditions, we conducted the semi *in situ* XPS characterization after treatment of the Ni_0.1_Cu_1_/CoO_*x*_ catalyst in an autoclave under 2 MPa H_2_ at 200 °C for 2 h (Fig. 8). The results demonstrated that the metal valences and oxygen states were very similar to the prepared Ni_0.1_Cu_1_/CoO_*x*_ catalyst (Fig. 4a–e). The outstanding CO_2_ hydrogenation performance of Ni_0.1_Cu_1_/CoO_*x*_ may be due to the synergy of the surface Co^2+^–O_v_–Co^2+^, Cu^0^/Cu^+^ and Ni–O–Co structures during the reaction.

**Fig. 8:**
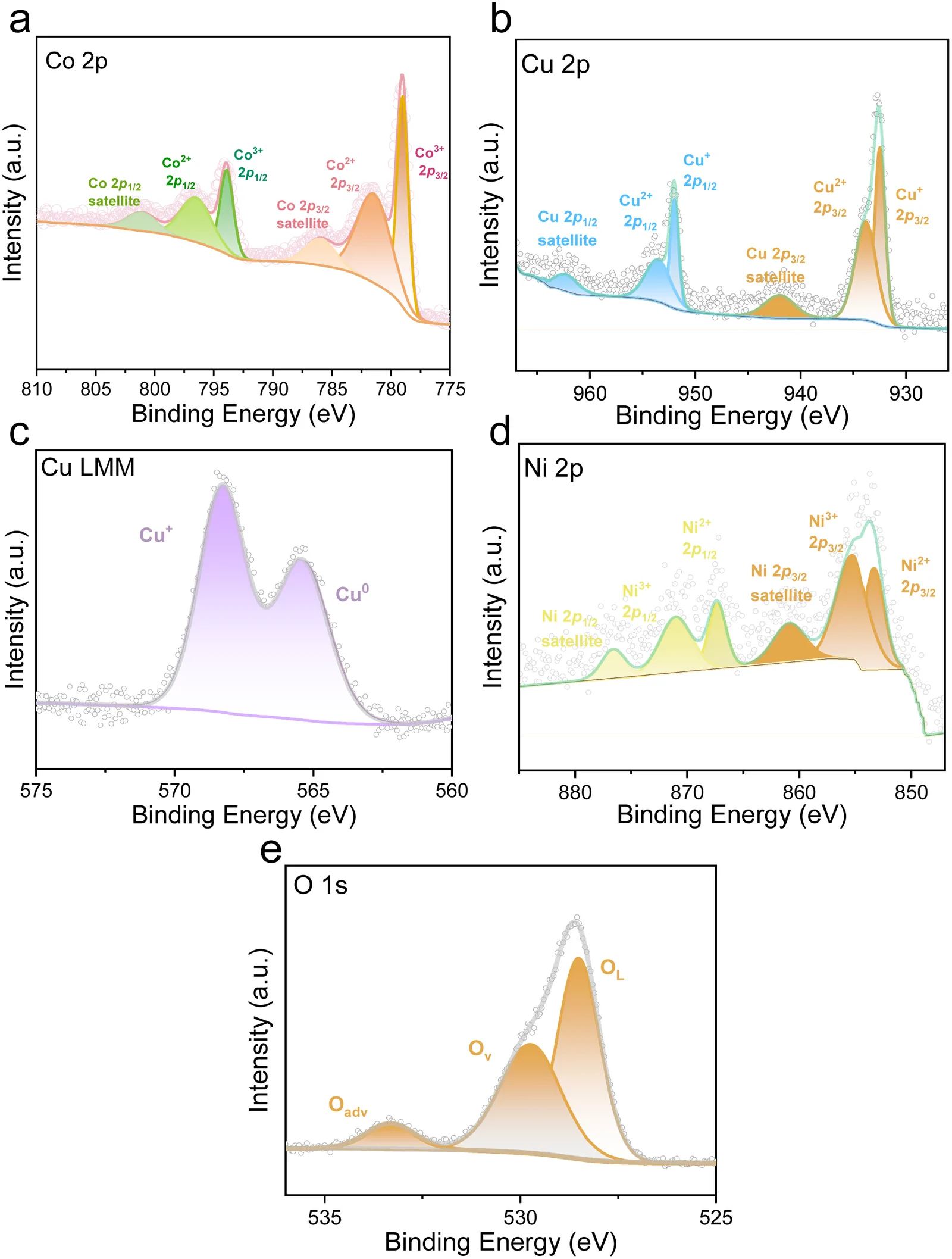
Semi *in situ* XPS spectra of Ni_0.1_Cu_1_/CoO_*x*_ pretreated with H_2_ at 200 °C for 2 h. (a) Co 2p, (b) Cu 2p, (c) Cu LMM, (d) Ni 2p, and (e) O 1s.

We further carried out the semi *in situ* study on the reaction of CO_2_ with the chemisorbed H atoms at different temperatures on the H_2_-pretreated catalyst surface. No obvious valence alteration of the metals (Co, Cu and Ni) and the oxygen states on the surface of the Ni_0.1_Cu_1_/CoO_*x*_ catalyst were observed (Fig. 9a–e). The reaction intermediates formed at different temperatures on the H_2_-pretreated catalyst were also analyzed (Fig. 9f). The peak at a binding energy of 292.2 eV corresponded to CO_2_ adsorbed on the catalyst surface, while the peak at a binding energy of 289.5 eV corresponded to the CO_3_^2−^ group. When the temperature increased from 50 to 200 °C, CO_2_ adsorbed on the surface of the Ni_0.1_Cu_1_/CoO_*x*_ catalyst was gradually transformed to the CO_3_^2−^ group, which might decompose into *CO. Then, *CO underwent further hydrogenation to form CH_3_O^−^, which corresponded to the peak at a binding energy of 285.9 eV.^63,68,69^ From 50 °C to 200 °C, the CH_3_O^−^ species continued to be reduced to hydrocarbons at 284.8 eV by the H atoms pre-adsorbed on the catalyst surface.^27^

**Fig. 9:**
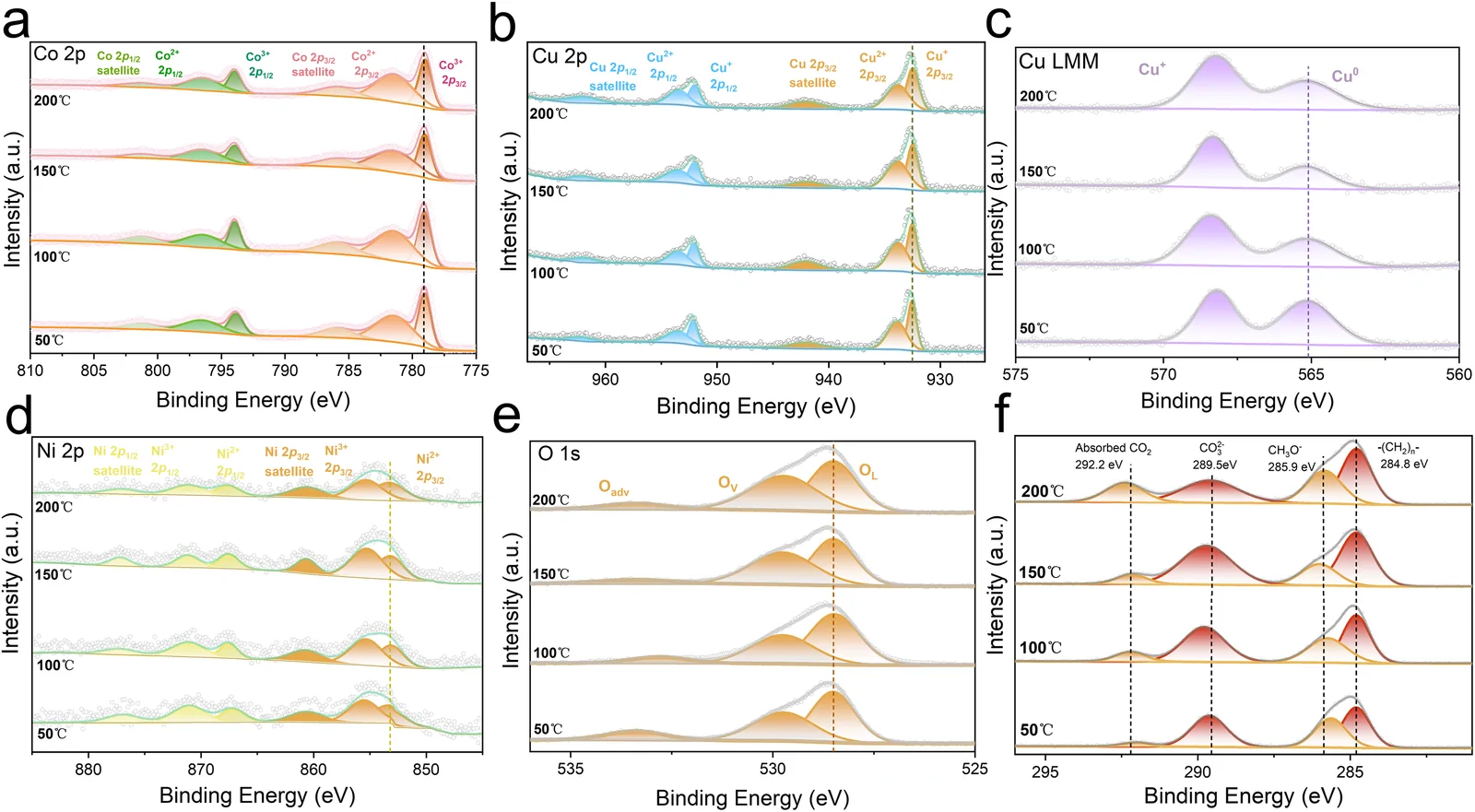
Semi *in situ* XPS spectra of Ni_0.1_Cu_1_/CoO_*x*_ after CO_2_ adsorption at different temperatures on the catalyst pretreated with H_2_ at 200 °C for 2 h. (a) Co 2p, (b) Cu 2p, (c) Cu LMM, (d) Ni 2p, (e) O 1s, and (f) C 1s.

Infrared spectroscopy is an important technique for studying surface adsorption of reactants and reactive intermediates generated *in situ*. *In situ* diffuse reflectance infrared Fourier transform spectroscopy (DRIFTS) characterization of CO_2_ hydrogenation was performed over a period of 0–90 min under 0.1 MPa CO_2_ at 180 °C over the Ni_0.1_Cu_1_/CoO_*x*_ catalyst, which was pre-treated with 10% H_2_/Ar at 250 °C for 2 h. The results of the *in situ* DRIFTS study are shown in Fig. 10.

**Fig. 10:**
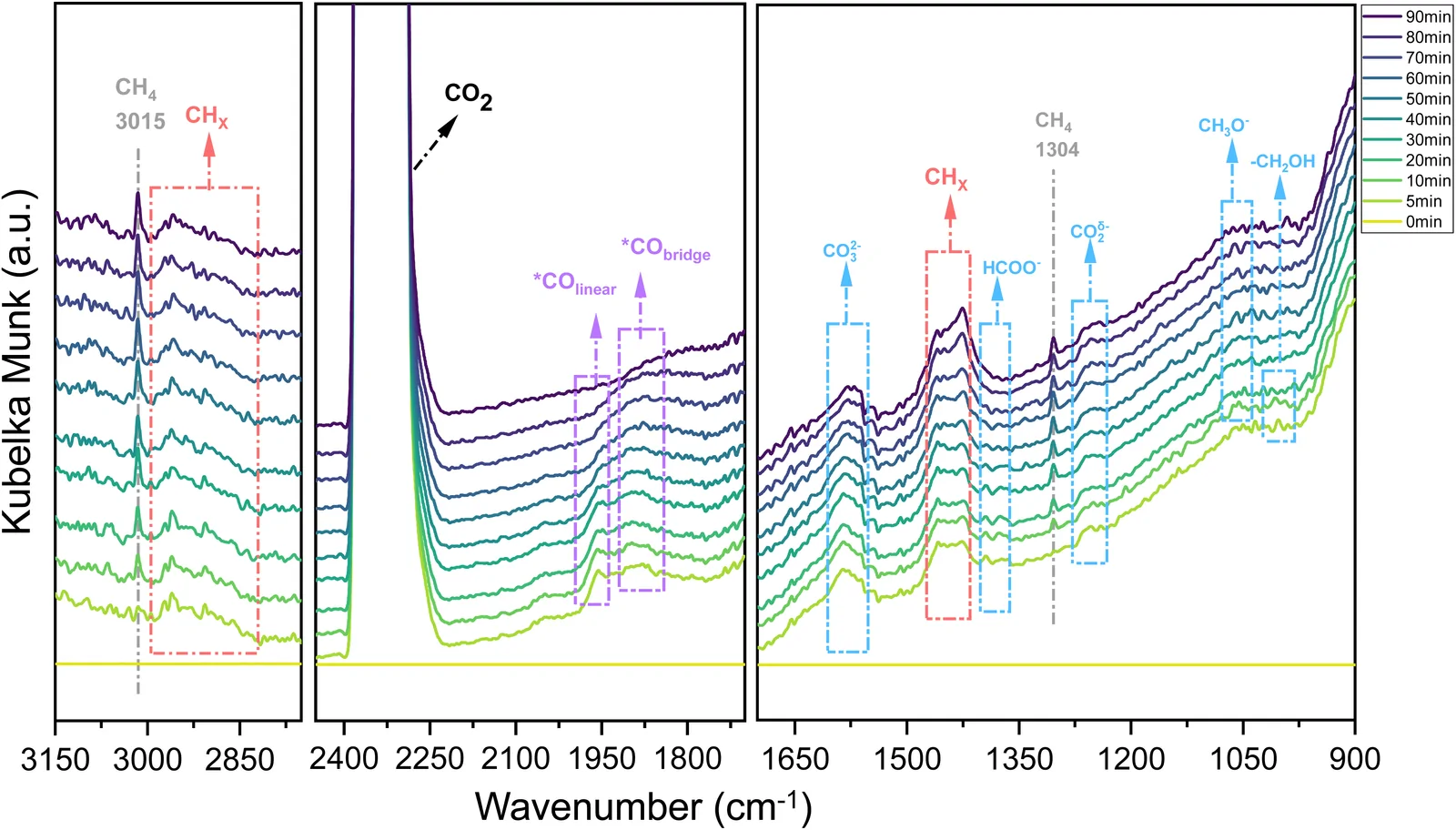
*In situ* DRIFT spectra during CO_2_ adsorption at 180 °C under a CO_2_ flow of 0.1 MPa over the Ni_0.1_Cu_1_/CoO_*x*_ catalysts pretreated with 10 vol% H_2_/Ar at 250 °C for 2 h.

It can be seen that CO_2_ was adsorbed on the catalyst surface and gradually reduced to CH_*x*_ and CH_4_, *via* CO_2_^*δ*−^, CO_3_^2−^, HCOO^−^, *CO, –CH_2_OH, and/or CH_3_O^−^ intermediates. At first, it was reduced to CO_2_^*δ*−^ by H atoms on the catalyst surface, as can be seen from the peaks at 1240–1270 cm^−1^. In addition, carbonate (CO_3_^2−^) peaks (1531–1614 cm^−1^) and HCOO^−^ infrared peaks (1366–1398 cm^−1^) were detected. Later, obvious CH_4_ peaks (3015 and 1304 cm^−1^) appeared from the fifth minute onwards. As hydrogenation progressed further, the CO_3_^2−^ peak strengthened, while the HCOO^−^ peak weakened. The spectrum shows clear infrared signal peaks of linear adsorption *CO (1939–1983 cm^−1^) and bridge adsorption *CO (1780–1920 cm^−1^).^70–73^ With increasing time, the *CO peaks in the adsorbed state gradually weakened, and the linear adsorption *CO peak gradually disappeared at 80 min, indicating their further reduction by the pre-adsorbed H atoms. With the conversion of *CO, the oxygenate species such as primary alcohol group (–CH_2_OH, 1007 cm^−1^) and/or methoxy group (CH_3_O^−^, 1059 cm^−1^) were observed, which is in agreement with the semi *in situ* XPS results (Fig. 9f).^27,74,75^ Meanwhile, the *CH_*x*_ peaks (2820–2990 cm^−1^ and 1415–1475 cm^−1^) and the CH_4_ signal increased continually, suggesting further hydrogenation of –CH_2_OH/CH_3_O^−^ species. The *CH_*x*_ species (*CH_2_ and *CH_3_) are the monomers for C–C coupling to form C_2+_ hydrocarbon products.^39,72,76^ No carboxylic acid, alcohol, or ether was detected in the reaction product (Fig. S15), indicating that –CH_2_OH and/or CH_3_O^−^ intermediates were finally converted into hydrocarbons.

We also conducted the reaction under a low pressure of 0.5 MPa (Fig. S17–S19). It can be observed that the catalyst still retains certain reactivity toward CO_2_ hydrogenation at low pressure, affording a considerable amount of C_2+_ hydrocarbon products. Therefore, the conclusions drawn from the *in situ* DRIFTS experiments are reasonable to deduce the reaction mechanism.

To better understand the active phase formed during the reaction and electronic structure, we performed a series of characterization studies on the spent catalyst after the reaction. We carried out XRD, HR-TEM, STEM-HAADF, EDS elemental mapping and XPS measurements on the spent Ni_0.1_Cu_1_/CoO_*x*_ catalyst. The XRD results reveal that the spent Ni_0.1_Cu_1_/CoO_*x*_ catalyst is mainly composed of Cu^0^ (PDF#04-0836), Co_2_C (PDF#05-0704), and a minor fraction of CoCO_3_ (PDF#11-0692) (Fig. S20). This indicates that Cu^0^ and Co_2_C are the active phases formed during the catalytic reaction, which is consistent with previous reports.^38^ The formation of the CoCO_3_ phase is likely attributed to the carbonation of cobalt oxides by the H_2_O–CO_2_ mixture in the reaction system, where water is a byproduct of the reaction.^77^ Meanwhile, as observed in Fig. S21, three distinct lattice spacings of 0.209 nm, 0.211 nm and 0.274 nm appear simultaneously, corresponding to the Cu^0^ (111), Co_2_C (111) and CoCO_3_ (104) crystal planes, respectively, which is consistent with the XRD results. The XPS spectral results demonstrated that the metal valences and oxygen states were very similar to the semi *in situ* XPS characterization results of the Ni_0.1_Cu_1_/CoO_*x*_ catalyst (Fig. S22). Notably, the corresponding Co–C signal peak at 284.2 eV can be observed in the XPS C 1s spectrum of the spent Ni_0.1_Cu_1_/CoO_*x*_ catalyst, corroborating the formation of the Co_2_C phase.^39*a*^ In addition, agglomeration of the catalyst after the reaction was not considerable, as observed in TEM images. The STEM-HAADF and EDS elemental mapping images reveal uniform spatial distributions of Co, Ni, Cu, C, and O elements on the spent Ni_0.1_Cu_1_/CoO_*x*_ catalyst. No obvious enrichment or depletion of any metal species is observed, demonstrating that segregation of Ni/Co/Cu species does not occur notably after the CO_2_ hydrogenation reaction.

According to the H_2_-TPR results, Ni doping favored the formation of Cu^0^/Cu^+^ active sites, which further facilitated the formation of the active phase Cu^0^ during the reaction. At the same time, Ni doping significantly promoted the reduction of Co oxides, which could further facilitate the formation of the active phase Co_2_C during the reaction. The Cu^0^ sites mainly promoted the reverse water gas shift reaction, whereas the active sites for carbon chain growth resided on Co species.^38,78^Fig. 5a also demonstrates that the presence of Co sites significantly increased the production of C_2+_ hydrocarbons.

Based on all of the results above, we proposed a plausible mechanism, which is shown in Fig. 11, including reaction pathways and active sites. This work follows the CO_2_-FTS mechanism, involving both RWGS and FTS steps.^19^ CO_2_ was first adsorbed and activated at the Cu^0^/Cu^+^ sites of CuO_*x*_, which then underwent several intermediate steps, such as carbonates and bicarbonates. Subsequently, the *CO intermediate was formed *via* the RWGS reaction.^79,80^ The *CO intermediate was further hydrogenated to produce –CH_2_OH, and/or CH_3_O^−^ species for subsequent FTS steps.^74,75^ Further hydrogenation at defective CoO_*x*_ sites with Co^2+^–O_v_–Co^2+^, Ni–O–Co and/or Co_2_C structures converted the –CH_2_OH and/or CH_3_O^−^ species into CH_*x*_ species,^62,81,82^ which underwent chain growth to produce C_2+_ hydrocarbons or direct hydrogenation to generate CH_4_.^38,83,84^

**Fig. 11:**
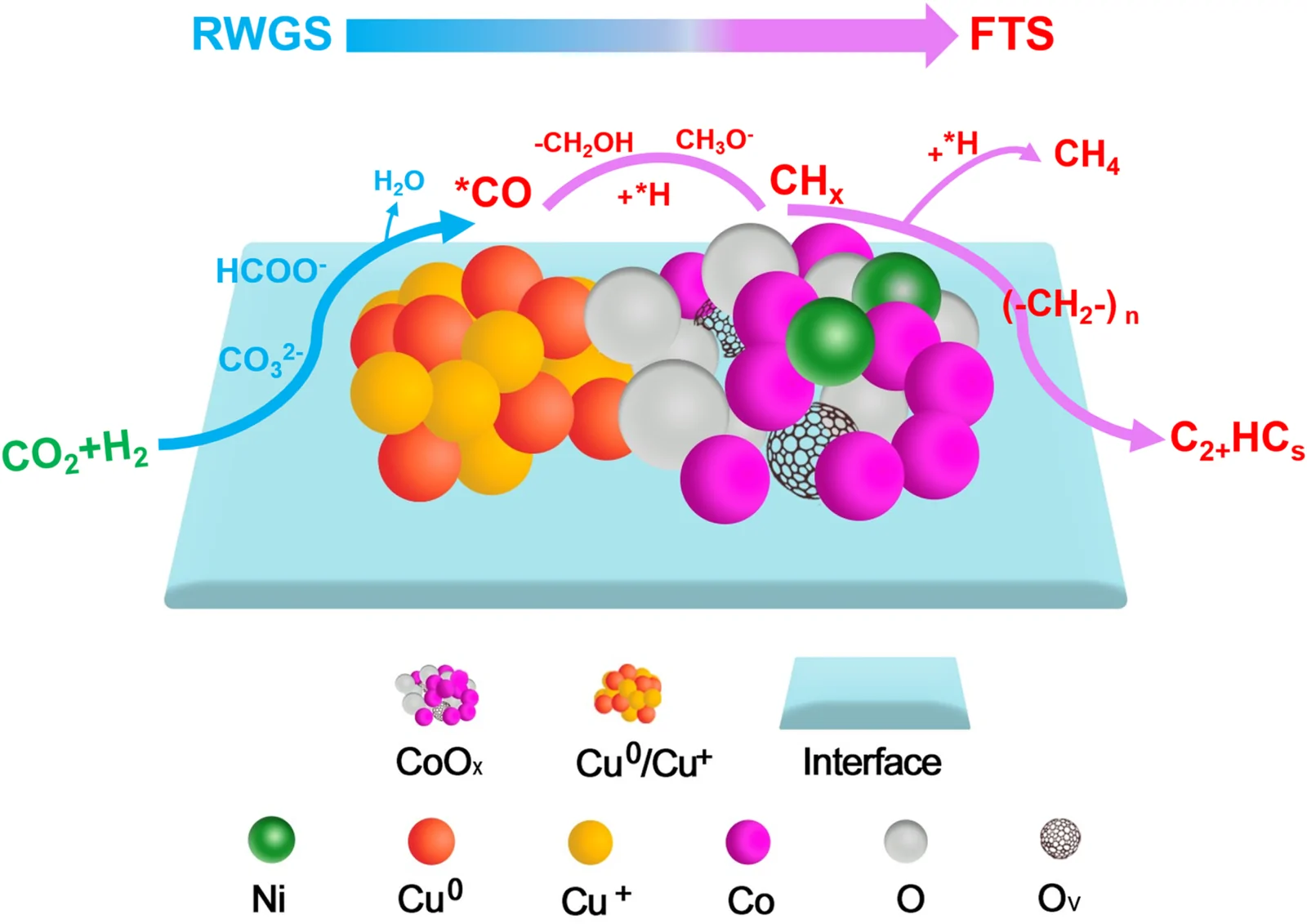
Proposed mechanism of CO_2_ hydrogenation over the Ni_0.1_Cu_1_/CoO_*x*_ catalyst.

## 3 Conclusions

In summary, we designed a novel and highly efficient Ni_0.1_Cu_1_/CoO_*x*_ catalyst for CO_2_ hydrogenation to produce C_2+_ hydrocarbons by doping Ni into the CuCo-based catalyst. Systematic characterization studies revealed that Ni doping modulated the catalyst's phase composition, enhanced strong interactions between Cu oxide and Co oxide species, and finely tuned the electronic structures of Cu and Co, which promoted the formation of a strongly coupled heterojunction structure (*i.e.*, Cu^0^/Cu^+^–CoO_*x*_). This facilitated the generation of abundant surface structures such as Co^2+^–O_v_–Co^2+^, Ni–O–Co, and Cu^0^/Cu^+^. Notably, the Ni_0.1_Cu_1_/CoO_*x*_ catalyst with a Ni doping ratio of Ni : Cu = 0.1 : 1 demonstrated impressive performance in the liquid–solid phase hydrogenation of CO_2_ using cyclohexane as the solvent at a low temperature of 200 °C. It exhibited a high C_2+_ hydrocarbon selectivity of 63.0% and a remarkable activity of 1.41 mmol g_cat_^−1^ h^−1^, ranking it among the most outstanding metal oxide-based catalysts reported. The mechanistic study demonstrated that the Ni-doped Cu^0^/Cu^+^–CoO_*x*_ interface played an important role in promoting C_2+_ hydrocarbon production. Moreover, the synergistic interactions between active Cu^0^/Cu^+^ sites and CoO_*x*_ species bearing Co^2+^–O_v_–Co^2+^, Ni–O–Co, and/or Co_2_C sites accounted for stable CO_2_ adsorption, activation, and efficient conversion. The active sites on the catalyst surface boosted the formation of key *CO and *CH_*x*_ species, and their transformation to C_2+_ hydrocarbons *via* C–C coupling. The present findings provide a practical approach for designing efficient transition-metal-based catalysts to enhance CO_2_ hydrogenation for producing C_2+_ hydrocarbons.

## Author contributions

C. Y., Q. Q. and B. H. developed the idea and designed the experiments. C. Y. performed the majority of the experiments. J. H. and L. Z. carried out the SEM and TEM tests and analyzed the data. J. G., Z. Z, Y. W., Y. Z. and X. S. took part in discussion of some data. C. Y. and B. Z. performed the infrared test. C. Y. wrote the first draft of the manuscript. Q. Q., B. H. and X. M. revised the manuscript. All authors discussed the results and commented on the manuscript.

## Conflicts of interest

All the contributing authors report no conflict of interests in this work.

## Note added after first publication

This article replaces the version published on 29th September, to ensure that the degrees Celsius symbol appears correctly in the figures.

## Supplementary Material

SC-OLF-D6SC05518D-s001

## Data Availability

The data supporting this paper have been included as part of the supplementary information (SI). Supplementary information: experimental procedures, SEM and TEM images, experimental chromatographic data, and other data. See DOI: https://doi.org/10.1039/d6sc05518d.
