# Supplementary material for: Boosting CO2 hydrogenation to C2+ hydrocarbons via phase modulation using a Ni-doped CuCo catalyst
Source: Chem Sci. 2026 Sep 29. Online ahead of print. doi: 10.1039/d6sc05518d (PMC13655791; doi:10.1039/d6sc05518d)
Supplement: Supplementary file 1 [file SC-OLF-D6SC05518D-s001.pdf]

Supplementary Information (SI) for Chemical Science.
This journal is © The Royal Society of Chemistry

Supporting Information

Experimental methods

1.1 Materials

$Ni(NO_3)_2·6H_2O$(99.99 %), cyclohexane (A. R. grade) were obtained from Sinopharm Chem. Reagent Co. Ltd. $Cu(NO_3)_2·3H_2O$(99.99 %), $Co(NO_3)_2·6H_2O$(99.99 %) were obtained from Innochem Technology Co., Ltd. Beijing. $Fe(NO_3)_3·9H_2O$(> 98 %), $Zn(NO_3)_2·6H_2O$ (99.99 %) were purchased from Alfa Aesar China Co., Ltd. $Ce(NO_3)_3·6H_2O$ (99.5 %), $H_2PtCl_6·6H_2O$(> 99.9 %), and 1,3-Dimethyl-2-imidazolidinone (DMI, 99 %) were bought from Aladdin. Na_2CO_3(> 99.8 %), N-methyl-2-pyrrolidone (NMP, 99 %), and methonal(99.9 %) were provided by J&K Scientific Ltd. CO_2(99.99 %) and H_2(99.99 %) were purchased from Beijing Analytical Instrument Company. Deionised H_2O was applied at all stages where water was needed. All the chemicals were utilized without any additional purification.

1.2 Catalyst preparation

Ni_nCu_1/CoO_x catalysts were prepared by co-precipitation. A typical procedure involved dissolving $Ni(NO_3)_2·6H_2O$ (0.024 g, 0.083 mmol), $Cu(NO_3)_2·3H_2O$ (0.2 g, 0.83 mmol), and $Co(NO_3)_2·6H_2O$ (1.45 g, 4.98 mmol) in 30 mL of water and stirring for 0.5 hours at room temperature. The mixed metal salt solution was slowly dropped into 100 mL of Na_2CO_3 solution (0.5 mol/L) at 50 °C under stirring within 1 h. After continued stirring at 50 °C for another 1 h, the mixture was allowed to age for 2 h at room temperature, and was then centrifuged to get the precipitate. The obtained precipitate was washed with 500 mL of deionized water and dried at 80 °C for 16 hours. Calcination of the obtained solid was carried out in a muffle furnace by heating it to 400 °C at a rate of 5 °C/min and maintaining this temperature for 6 hours, and then the catalyst was cool down naturally to room temperature. Subsequently, H_2/Ar (10 vol.%) was used to reduce the catalyst by heating it to 250 °C at a rate of 10 °C/min and maintaining this temperature for 2 hours. The collected catalyst was named as $Ni_{0.1}Cu_1/CoO_x$. By similar method, we also prepared a series of catalysts with different Ni content, Ni_nCu_1/CoO_x (n = 0.1, 0.25, 0.5, 1, and 2.5), where n denotes the mole ratio of Ni to Cu in the catalyst. The molar ratio of Cu and Co (1:6) in the above catalysts remained constant.

In order to investigate the effects of the three components of Ni, Cu, and Co on the catalysts, we also prepared CoO_x, NiO_x, Cu/CoO_x, Ni/CuO_x and Ni/CoO_x catalysts of different metal ratios, with the similar preparation procedure as that of Ni_nCu_1/CoO_x, except that the precursors of the corresponding missing metal elements were not added. In addition to the Ni_nCu_1/CoO_x catalysts, in order to compare the effects of different metal doping on the Cu/CoO_x catalysts, we also prepared $Pt_{0.1}Cu_1/CoO_x$, $Fe_{0.1}Cu_1/CoO_x$, $Ce_{0.1}Cu_1/CoO_x$, and $Zn_{0.1}Cu_1/CoO_x$ catalysts, and the preparation procedure was the same as that of $Ni_{0.1}Cu_1/CoO_x$.

The recycling test

The $Ni_{0.1}Cu_1/CoO_x$ composite could be recycled and reused. After each run, the catalyst was separated from the reaction mixture via centrifugation, washed with water for several times, and dried at 80 °C overnight. The catalyst was then used directly in the subsequent reaction.

1.3 Catalyst characterization

X-ray diffraction (XRD) patterns were obtained using a Rigaku D/max 2500 X-ray diffractometer, operating at 40 mA and 30 kV with Cu Kα radiation at a scanning speed of 5° min^{-1} over the 2θ ranging from 15°-85°. Scanning electron microscopy (SEM) images were taken using a Hitachi S-4800 electron microscope. Transmission electron microscopy (TEM) and high-resolution TEM (HRTEM) images were acquired utilizing a JEOL JEM-2100F microscope, and the energy-dispersive X-ray spectroscopic (EDS) elemental distribution mapping images were obtained at 200 kV to analyze the elemental distribution. The particle size distribution was determined using the Scherrer formula according to the TEM images. The N_2 adsorption/desorption experiments were measured on Micromeritics ASAP 2020 M system, and the surface area data were calculated based on the Brunauer-Emmett-Teller (BET) model. Inductively Coupled Plasma Optical Emission Spectrometer (ICP-OES) analysis was conducted on the Thermo iCAP RQ instrument to determine the composition of the catalysts.

X-ray photoelectron spectroscopy (XPS) spectra were recorded on an ESCALab 220I-XL electron spectrometer from VG Scientific using Al Kα radiation with a hemispherical energy analyzer. All measured binding energies were calibrated using the C 1 s level (284.8 eV) as a reference. X-ray absorption fine structure (XAFS) measurements were carried out on 1W1B beamline of Beijing Synchrotron Radiation Facility (BSRF). All of the obtained spectra were calibrated and normalized using the Athena program. Fourier transform (FT) and wavelet transform (WT) extended X-ray absorption fine structure (EXAFS) spectra were obtained via the Athena and Hama Fortran programs, respectively.

The H_2 temperature programmed reduction (H_2-TPR) was performed in an AutoChem II2920 instrument equipped with a thermal conductivity detector (TCD). 100 mg of fresh catalyst was pretreated in Ar (30 mL/min) to 300 °C with a heating rate of 10 °C/min and kept for 0.5 h, then it was cooled to 50 °C and the TPR profile was recorded from 50 to 1000 °C at a ramping rate of 10 °C/min with the gas flow 10 vol. % H_2/Ar (30 mL/min). The CO_2 temperature programmed desorption (CO_2-TPD) experiments were also carried out by the AutoChem II2920 instrument. Before CO_2-TPD experiments, 100 mg sample was pretreated in He (30 mL/min) to 200 °C with a heating rate of 10 °C/min and kept for 1 h, then it was cooled to 50 °C, followed by CO_2 (30 mL/min) adsorption at 50 °C for 2 h. After purging the extra CO_2 with He (30 mL/min) for 1 h, the CO_2 desorption was conducted from 50 °C to 1000 °C in the He atmosphere with a heating rate of 10 °C/min and the desorption profile was collected every second.

The semi-in situ XPS measurement was aided by a special vacuum seal sample unit to transfer the sample prepared in the glove box. To get the state of the catalyst surface under reaction condition, the $Ni_{0.1}Cu_1/CoO_x$ was treated with 2 MPa of H_2 at 200 °C for 2 h, then the reactor was cooled and the H_2 was released, the sample was prepared in the glove box, and transferred to the sample chamber of an XPS spectrometer using the sample unit. To study the CO_2 adsorption on the catalyst surface, $Ni_{0.1}Cu_1/CoO_x$ was pretreated with 2 MPa of H_2 at 200 °C for 2 h. The reactor was cooled, and the H_2 was released and replaced with 0.7 MPa of CO_2 three times. Then the reactor was charged with 2 MPa of CO_2 and kept at the specified temperature for 1 h. Finally, the reactor was cooled to room temperature, and the gases were released. The sample was prepared in the glove box, and transferred to the sample chamber of an XPS spectrometer using the sample unit. To reduce the loss of adsor-bates during the XPS analysis, the sample chamber was kept at ultra-low temperature using liquid nitrogen.

The in situ diffuse reflectance infrared Fourier transform spectroscopy (DRIFTS) spectra were recorded with a FOLI10-R-T infrared spectrometer equipped with a DLaTGS detector supplied by INSA Co. Ltd. The measurements were conducted at 0.1 MPa in diffuse reflectance mode at a resolution of 4 cm^{-1}, with a scanning speed of one spectrum per minute. Before CO_2 adsorption, the catalyst underwent treatment in 10 vol.% H_2/Ar at 250 °C for 2 h. After the catalyst was cooled to 180 °C, the background spectrum was recorded. Subsequently, pure CO_2 was introduced into the in situ reaction cell, and spectra were continuously collected until no further changes were observed.

1.4 Catalytic reaction

The catalytic reactions were carried out in a 16 mL stainless steel reactor with a Teflon lining. In a typical experiment, a certain amount of catalyst and solvent were added to the reactor. At room temperature, the reactor was purged three times with 0.7 MPa CO_2 in order to remove the air in the reactor. Then CO_2 and H_2 with specific pressure were inflated into the reactor at room temperature, and the reactor was heated to a constant temperature to start the reaction. After the reaction was completed, the reactor was fully cooled in an ice-water bath and the residual gas was released slowly and collected in a gasbag. The gaseous samples (C1 products and CO_2) were analyzed and quantified by a gas chromatograph (GC, Agilent 7890B) equipped with a packed column (Shin Carbon ST, 2 mm in diameter and 2 m in length) and a TCD detector using argon as the carrier gas. The larger gaseous products (C2~C5) were analyzed and quantified by a GC (Agilent 8890) instrument equipped with a flame ionization detector (FID) and a HP-plot Al_2O_3 column using N_2 as the carrier gas. The liquid mixture was analyzed by a gas chromatograph (GC, Agilent 7890 B) equipped with a flame ionization detector (FID) and a DB-5 capillary column using toluene as an internal standard. The liquid products were identified using GC-MS (Agilent-7890B-5977A) as well as by comparing the retention times with the standards in the GC traces.

The conversion of CO_2 was calculated from the amount of reacted CO_2 divided by the total CO_2 added to the reactor. The total amount of CO_2 charged into the reactor could be obtained by weighing, and the reacted CO_2 was known from the amount of total carbon in the product.

The selectivity of C_{2+} hydrocarbons were calculated by dividing the molar amount of C atoms in C_{2+} hydrocarbons by the molar amount of C atoms of all hydrocarbon products. The activity of C_{2+} hydrocarbons referred to the mmol of C atoms in C_{2+} hydrocarbons produced per unit mass of catalyst per unit time. The selectivity of CO was obtained by dividing the molar mount of CO by the amount of CO_2 consumed during the reaction.

The chain growth probability (α) was calculated according to Anderson−Schulz−Flory distribution:

$$\left(\frac{W_n}{n}\right) = (1-\alpha)^2\alpha^{(n-1)}$$

where n is the carbon number of products, Wn is the mass fraction of the hydrocarbons with a carbon number of n, and α is the chain growth probability.

The equation can be edited as

$$\mathrm{Ln}\left(\frac{W_n}{n}\right) = (n-1)\,\mathrm{Ln}\,\alpha + 2\,\mathrm{Ln}(1-\alpha)$$

Plotting Ln(Wn/n) versus n, and the chain growth probability (α) can be obtained by calculating the slope (Lnα).

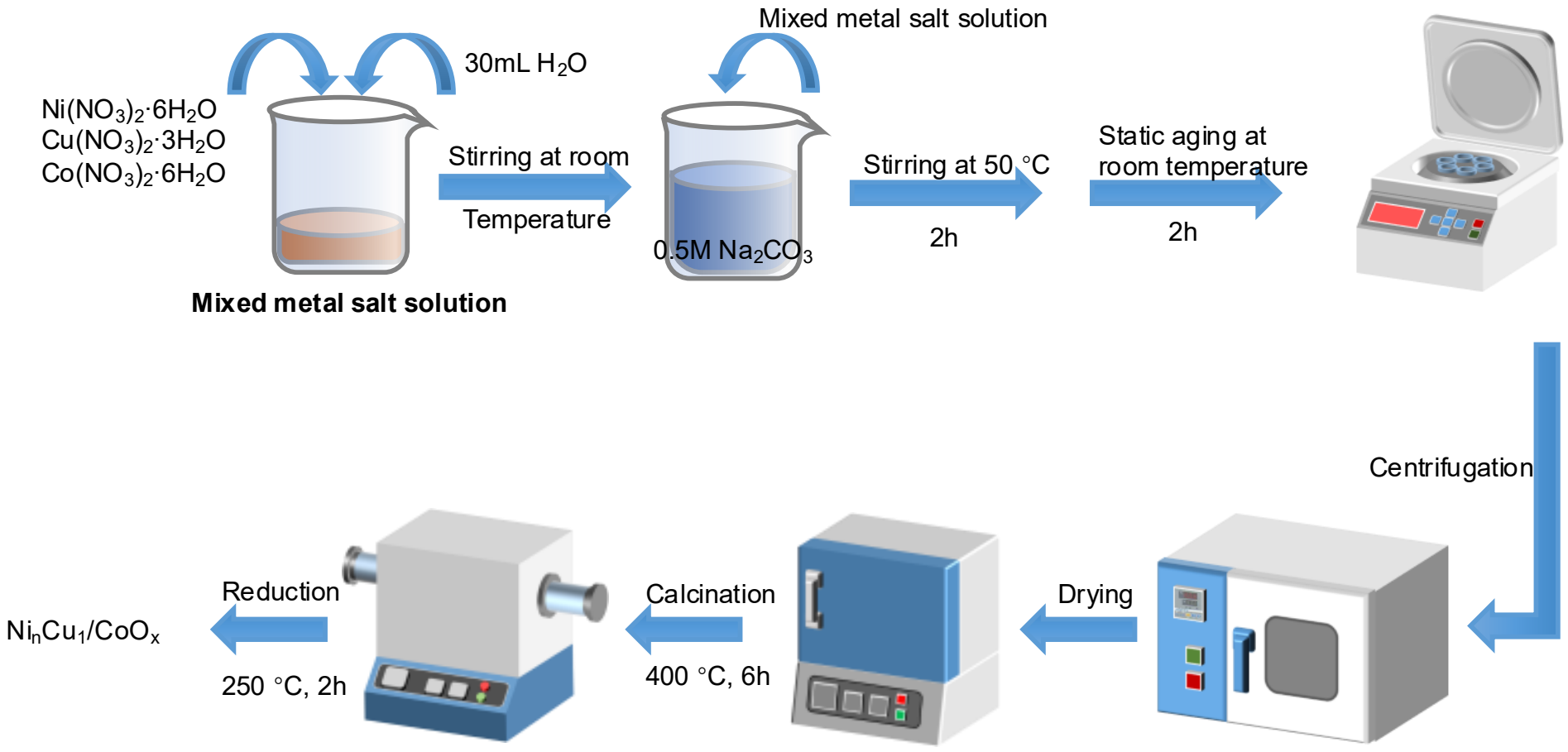

Fig. S1. Schematic illustration of the synthesis of Ni_nCu_1/CoO_x catalyst.

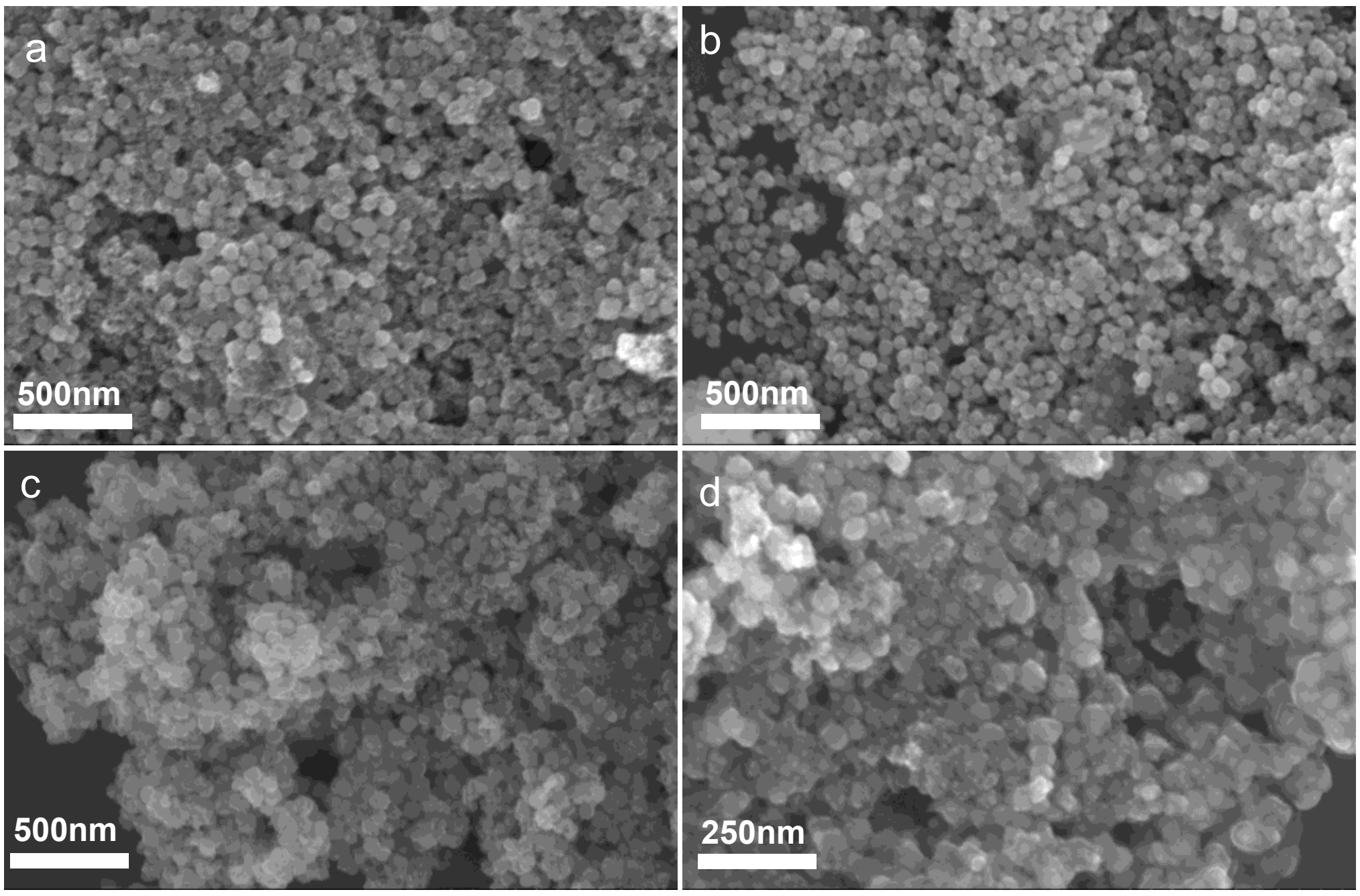

Fig. S2. SEM images obtained at different stages of $Ni_{0.1}Cu_1/CoO_x$ preparation: (a) the precipitate, (b) after calcination, (c,d) after reduction.

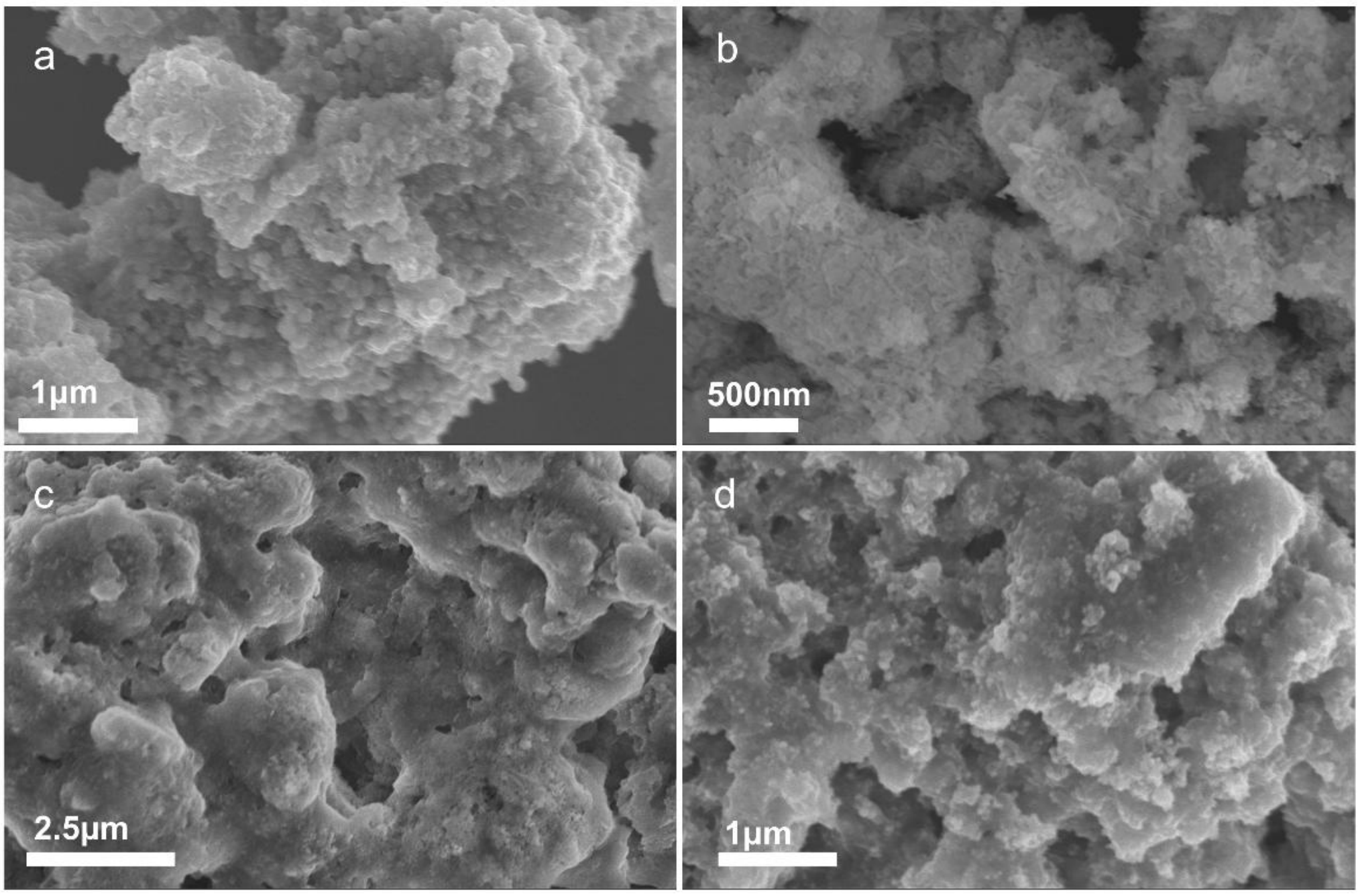

Fig. S3. SEM images of the prepared materials with different compositions: (a) $Ni_{0.5}Cu_1/CoO_x$, (b) $Ni_{2.5}Cu_1/CoO_x$, (c) Ni/CoO_x, (d) Cu/CoO_x.

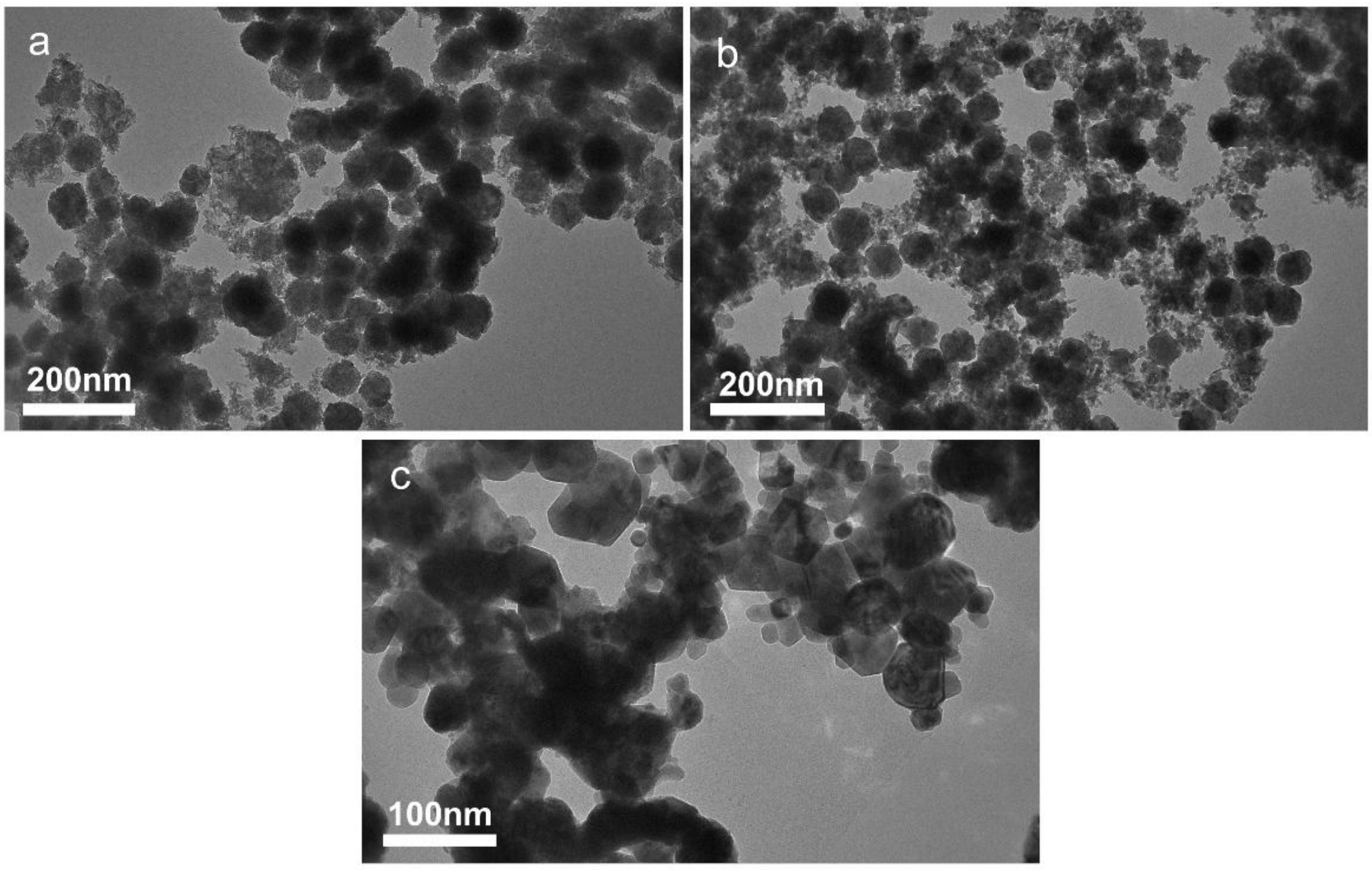

Fig. S4. TEM images obtained at different stages of $Ni_{0.1}Cu_1/CoO_x$ preparation: (a) the precipitate, (b) after calcination, (c) after reduction.

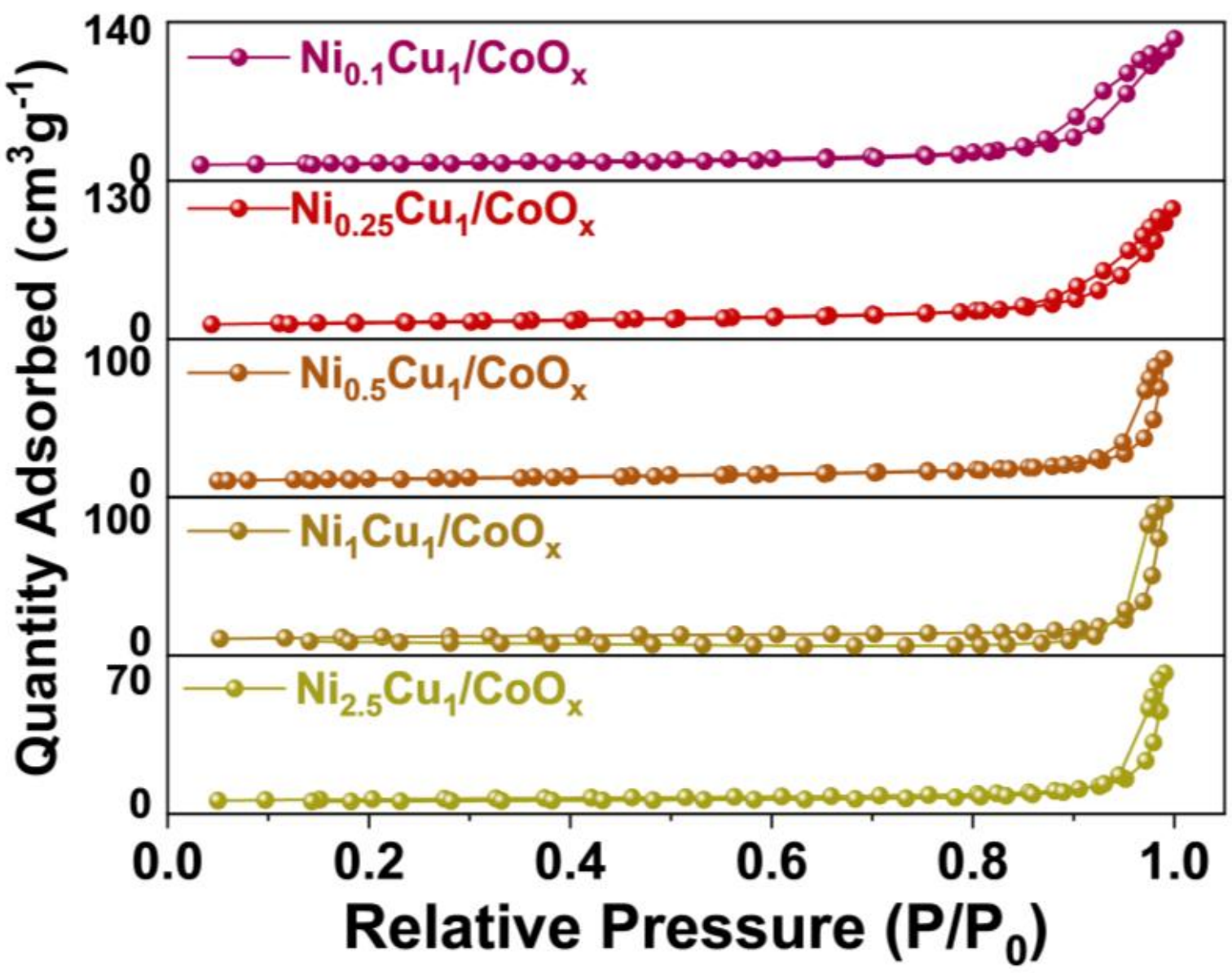

Fig. S5. The N_2 adsorption/desorption isothermal curves of the Ni_nCu_1/CoO_x catalysts.

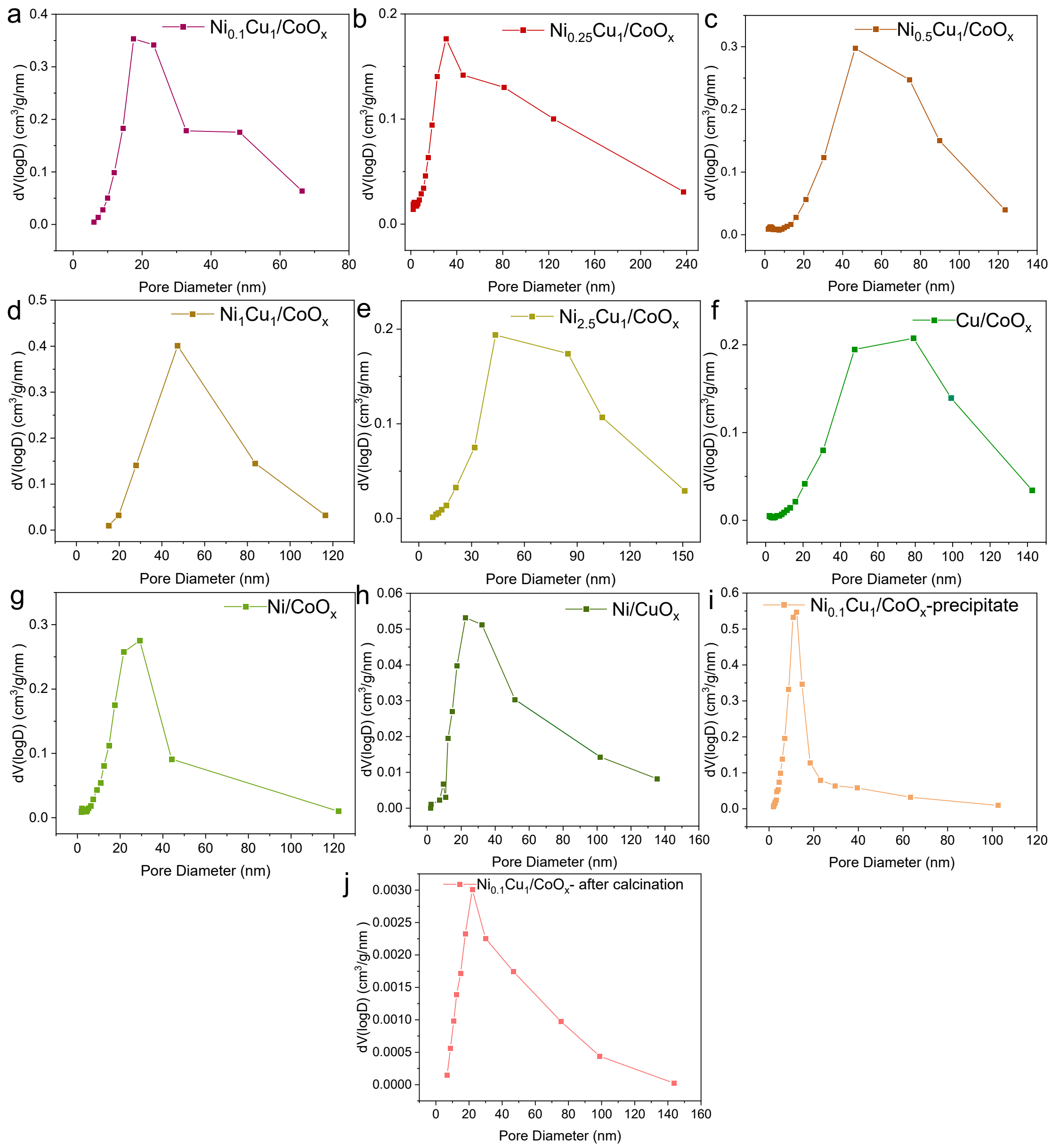

Fig. S6. Pore size distribution curves of all catalysts.

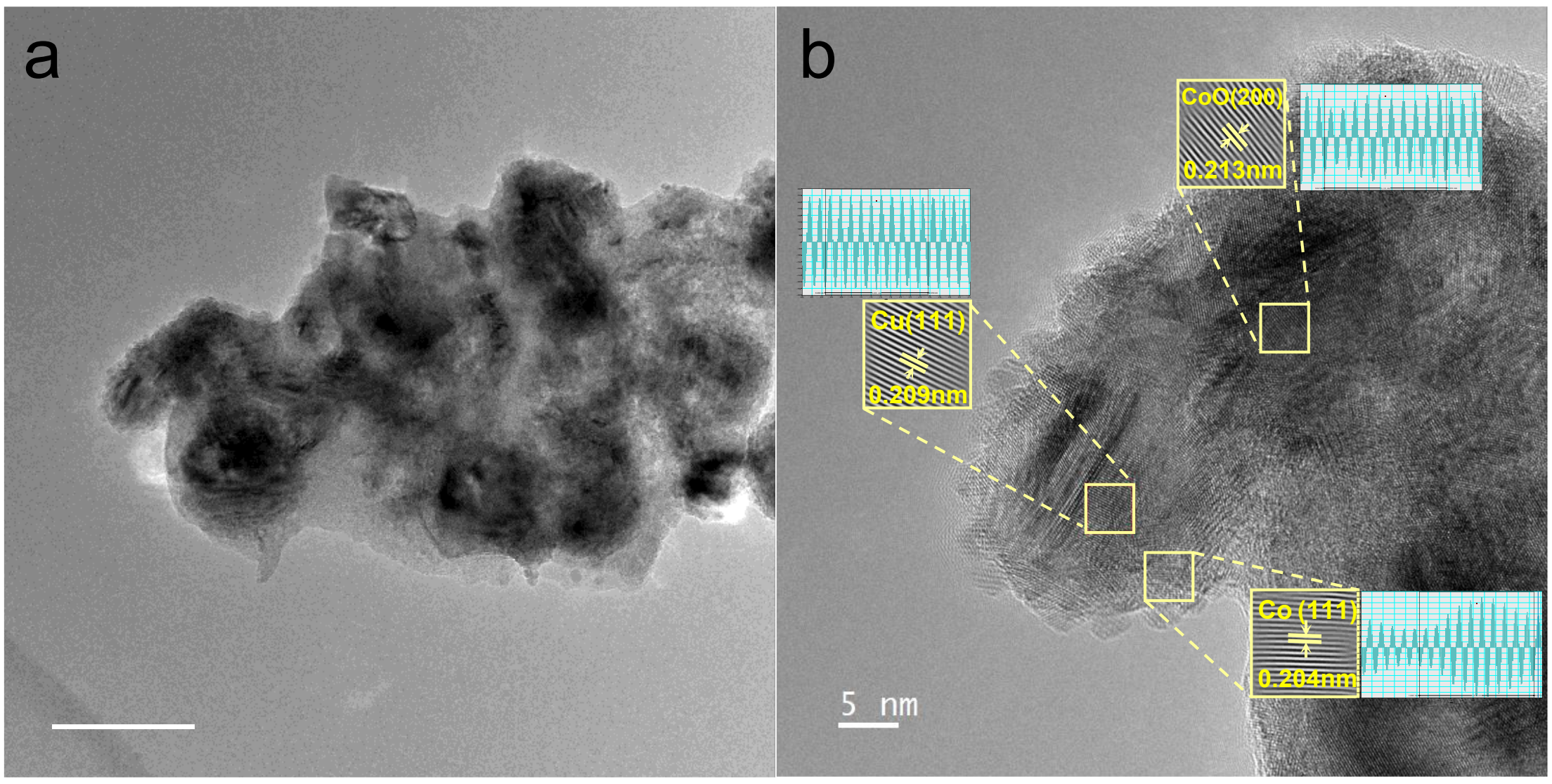

Fig. S7. (a), (b) High Resolution (HR)HR-TEM images of Cu/CoO_x including its inverse fast Fourier transform(IFFT) images and lattice fringe distances (Insets).

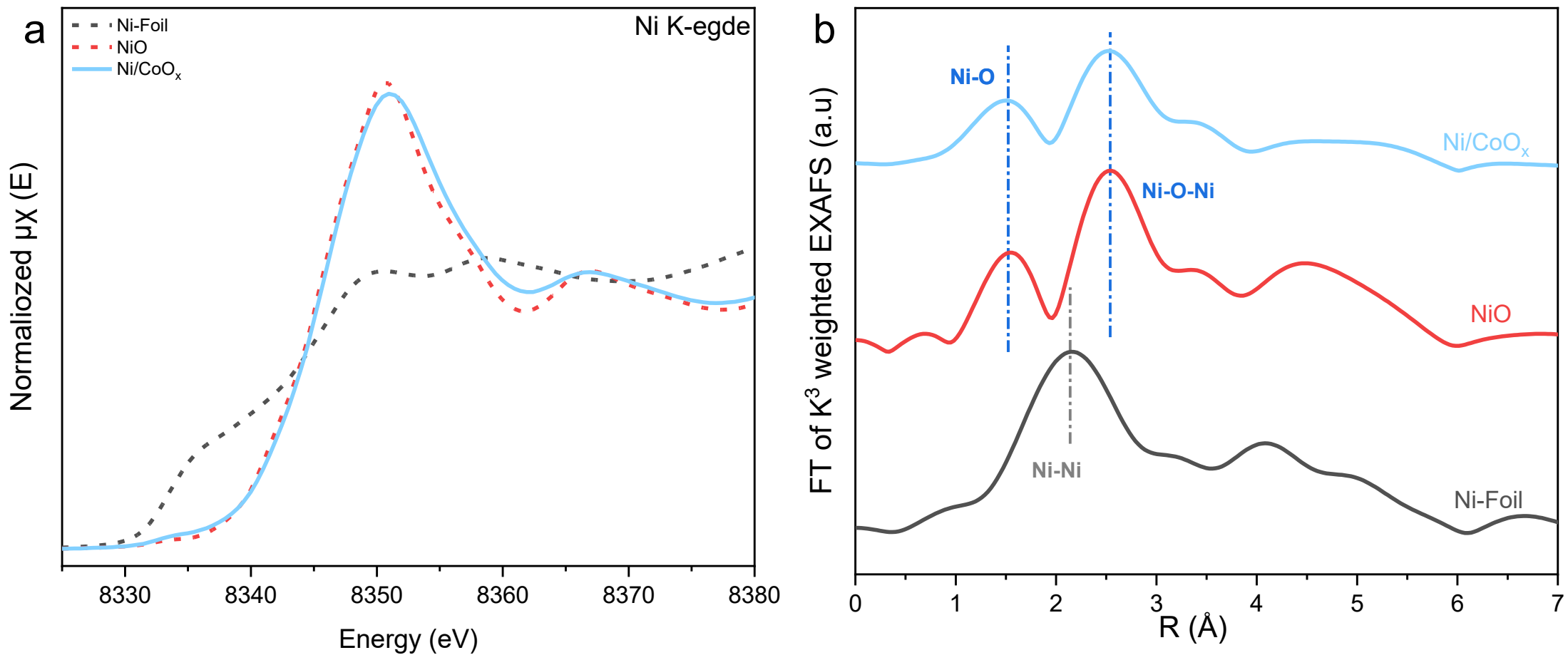

Fig. S8. (a) Normalized Ni K-edge XANES spectra, (b) Fourier transform (FT) magnitude k^3-weighted Ni K-edge EXAFS spectra of Ni/CoO_x catalyst.

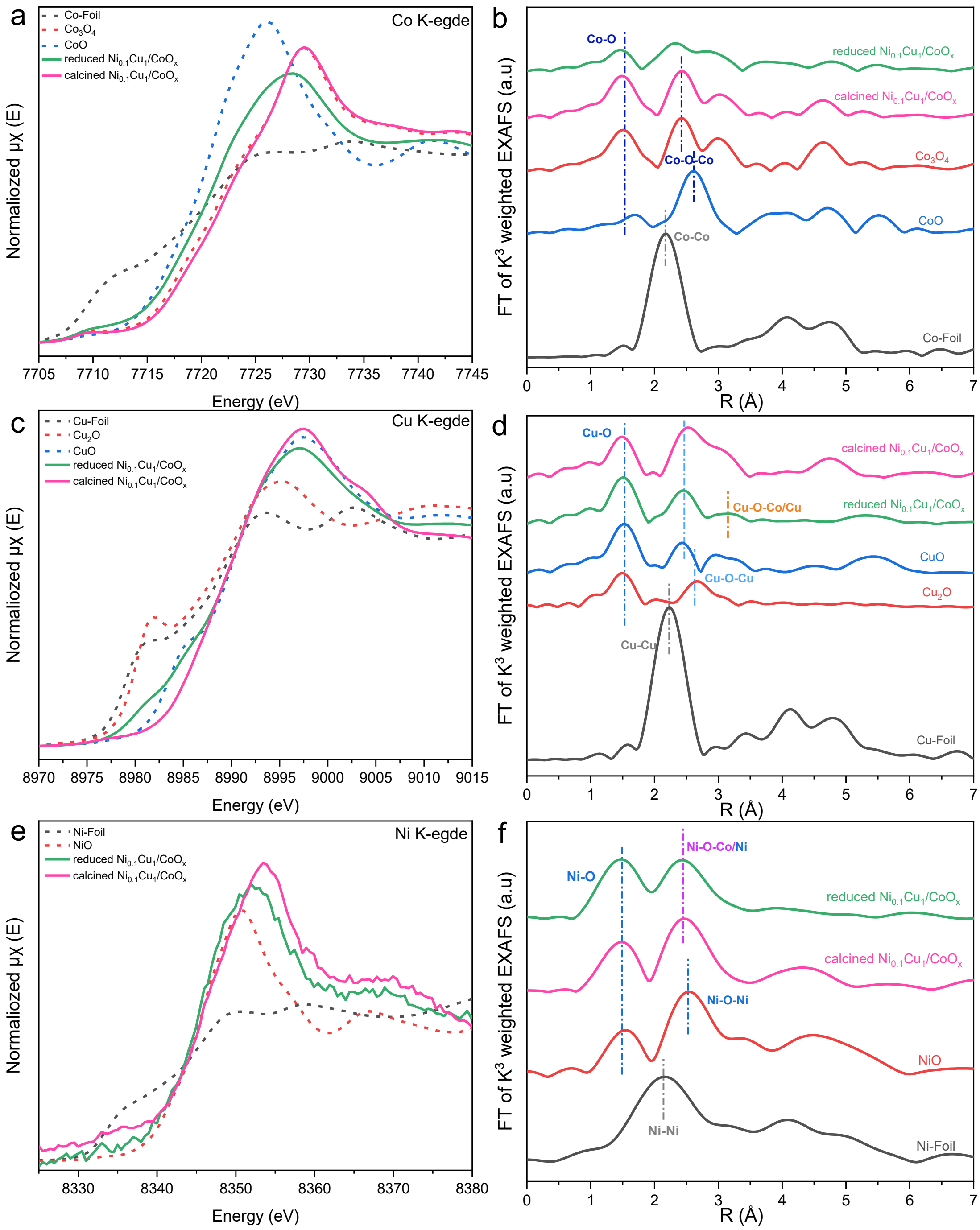

Fig. S9. (a) Normalized Co K-edge XANES spectra, (b) Fourier transform (FT) magnitude k^3-weighted Co K-edge EXAFS spectra of different states of $Ni_{0.1}Cu_1/CoO_x$ catalysts. (c) Normalized Cu K-edge XANES spectra, (d) FT magnitude k^3-weighted Cu K-edge EXAFS spectra of different states of $Ni_{0.1}Cu_1/CoO_x$ catalysts. (e) Normalized Ni K-edge XANES spectra, (f) FT magnitude k^3-weighted Ni K-edge EXAFS spectra of different states of $Ni_{0.1}Cu_1/CoO_x$ catalysts.

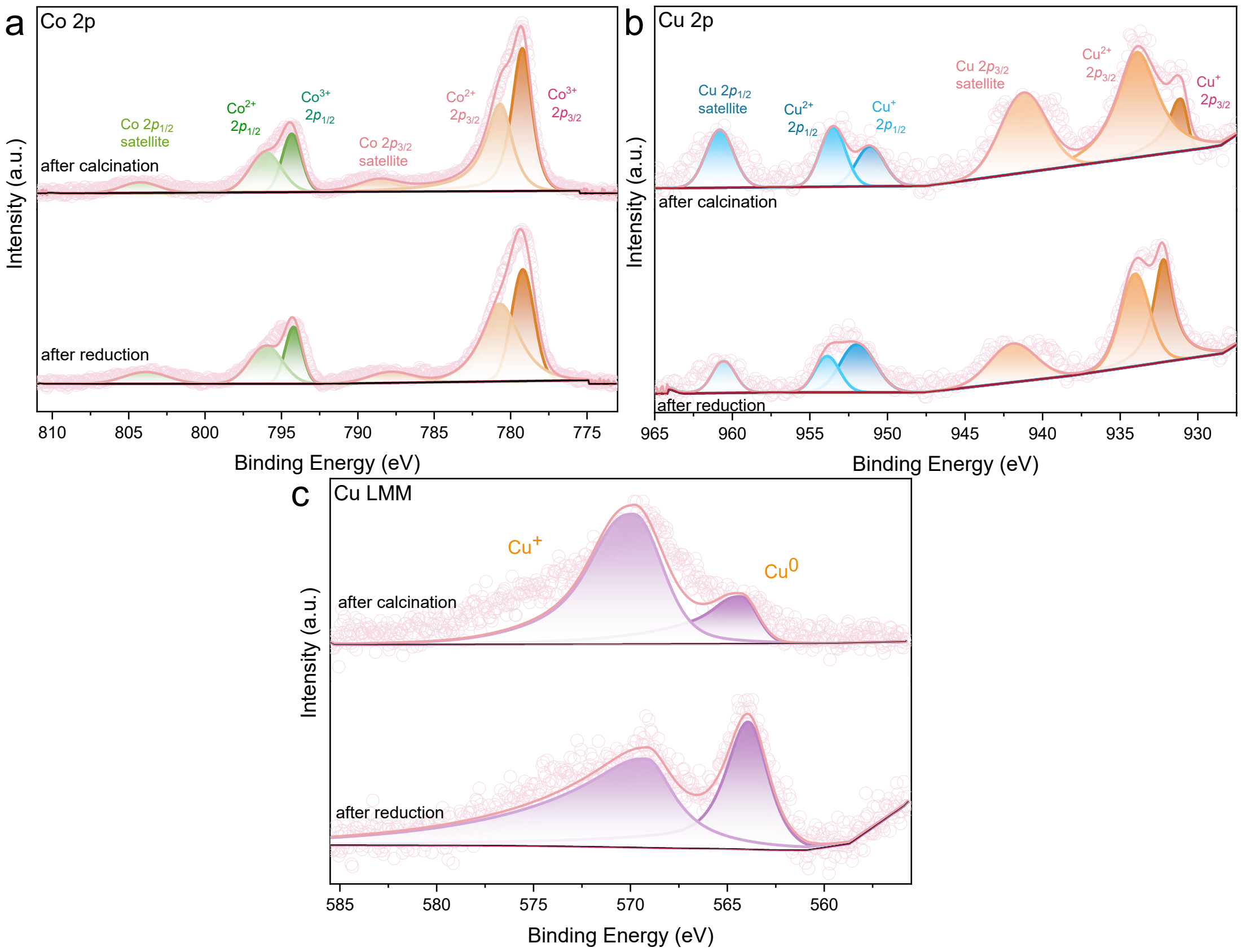

Fig. S10. XPS spectra of (a) Co 2p peaks, (b) Cu 2p peaks (c) Cu LMM peaks of different states of $Ni_{0.1}Cu_1/CoO_x$ catalysts.

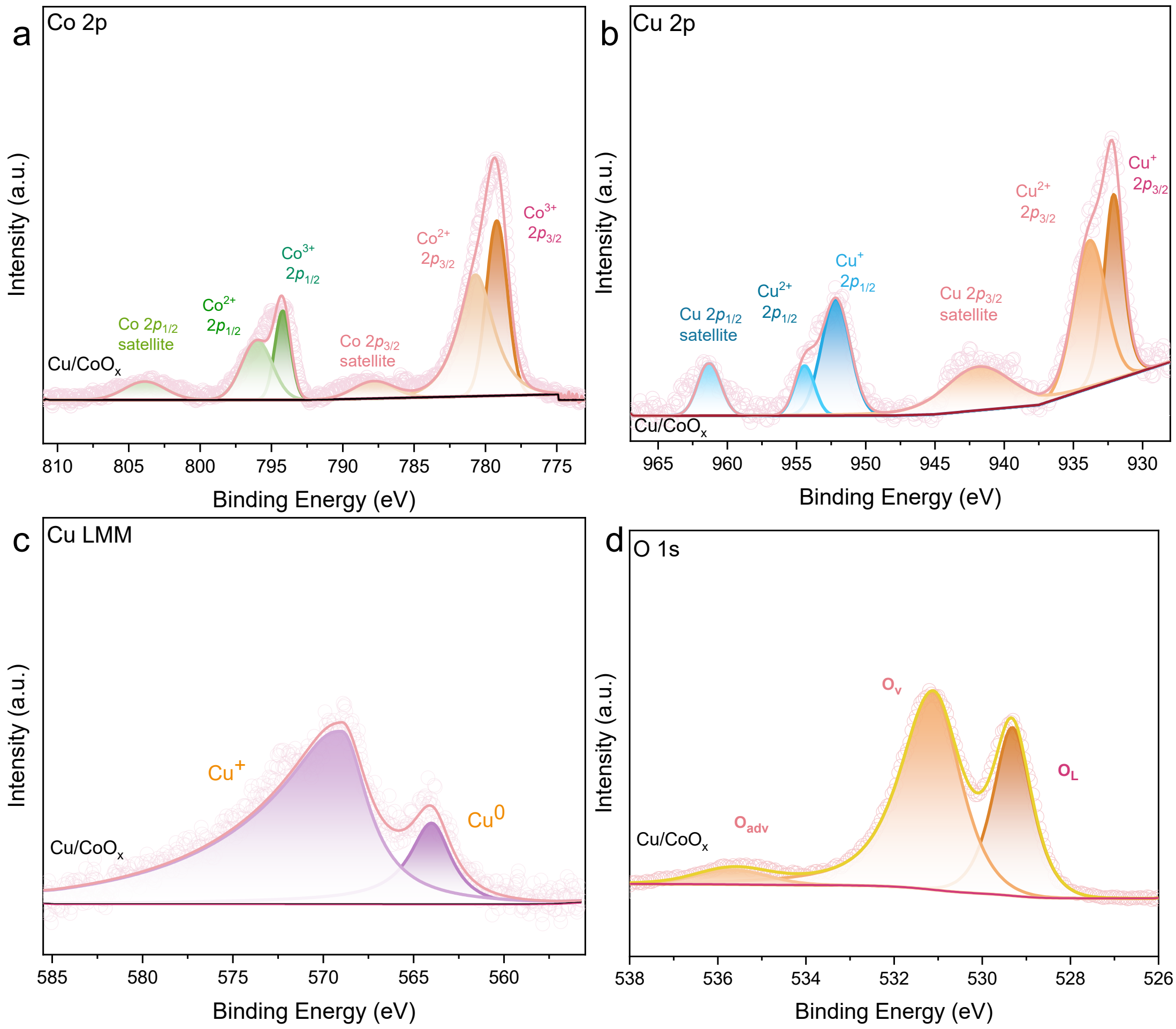

Fig. S11. XPS spectra of (a) Co 2p peaks, (b) Cu 2p peaks, (c) Cu LMM peaks, and (d) O 1s peaks of Cu/CoO_x catalysts.

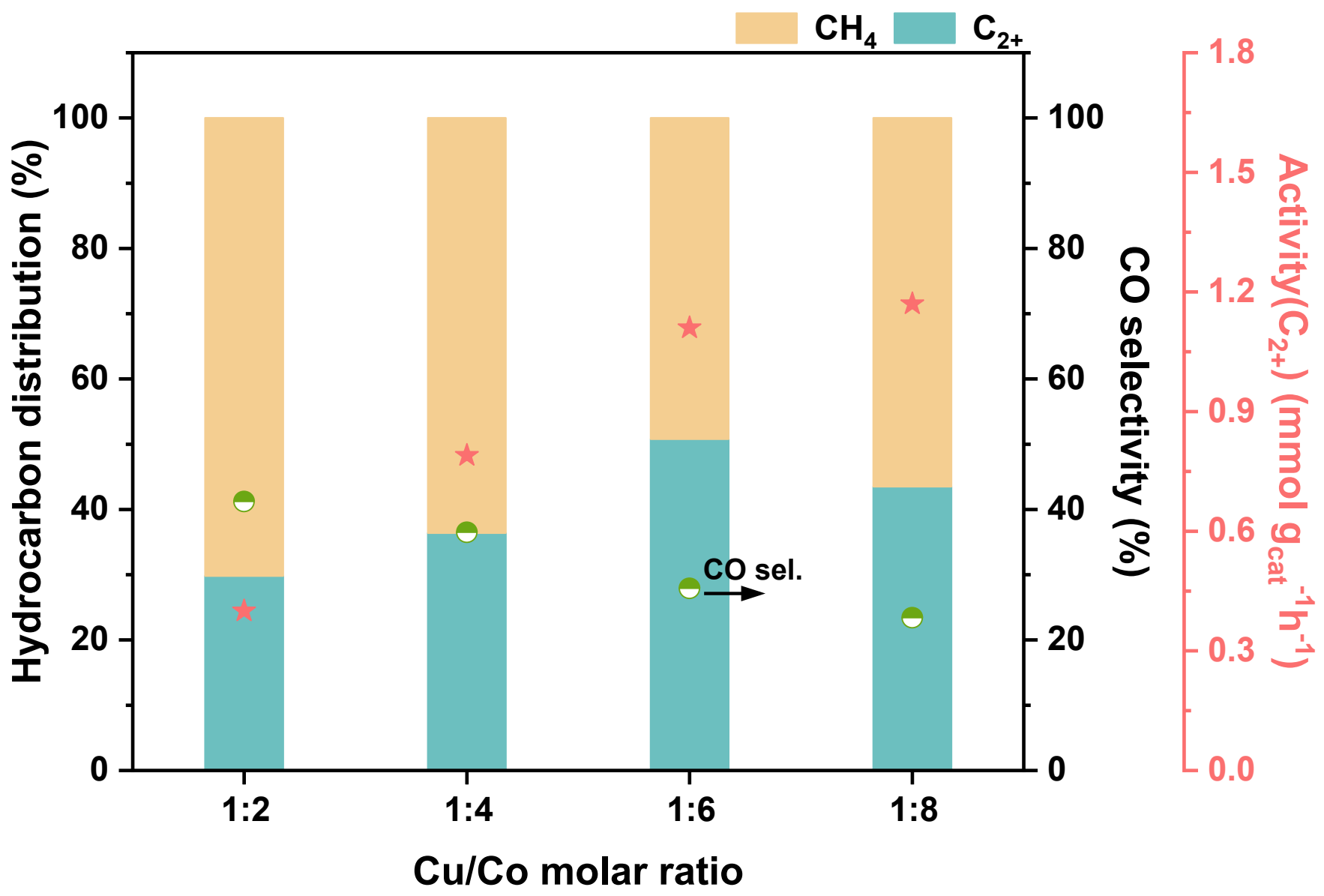

Fig. S12. CO_2 hydrogenation performance over Cu/CoO_x catalysts with different Co/Cu mole ratios. Reaction conditions: catalyst 50 mg and 2 mL of Cyclohexane, initial pressure 8.0 MPa (CO_2/H_2=1:1), 12 h, 200 °C.

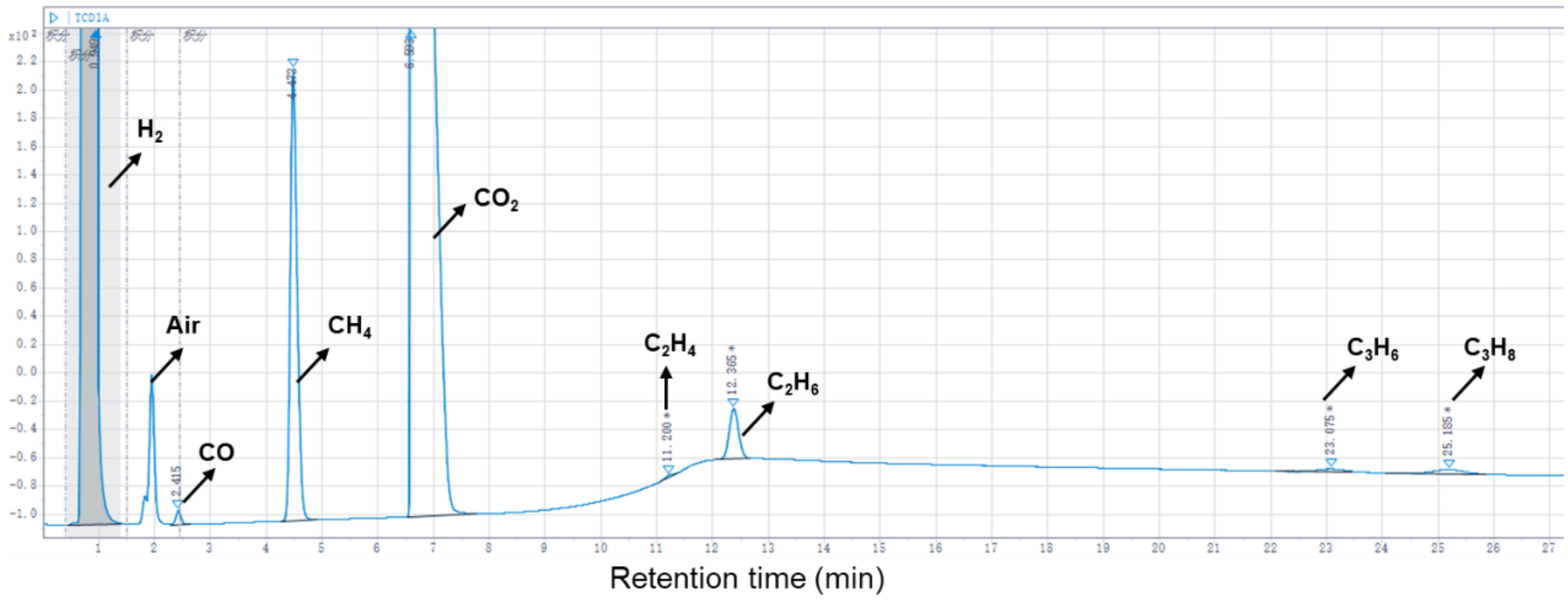

Fig. S13. The GC graph of the gaseous sample after CO_2 hydrogenation over the $Ni_{0.1}Cu_1/CoO_x$ catalyst at the optimal condition, which was obtained by a gas chromatograph (GC, Agilent 7890B) equipped with a packed column (Shin Carbon ST, 2 mm in diameter and 2 m in length) and a TCD detector using argon as the carrier gas.

Note: The C1 products and CO_2 in gaseous form were determined by this GC.

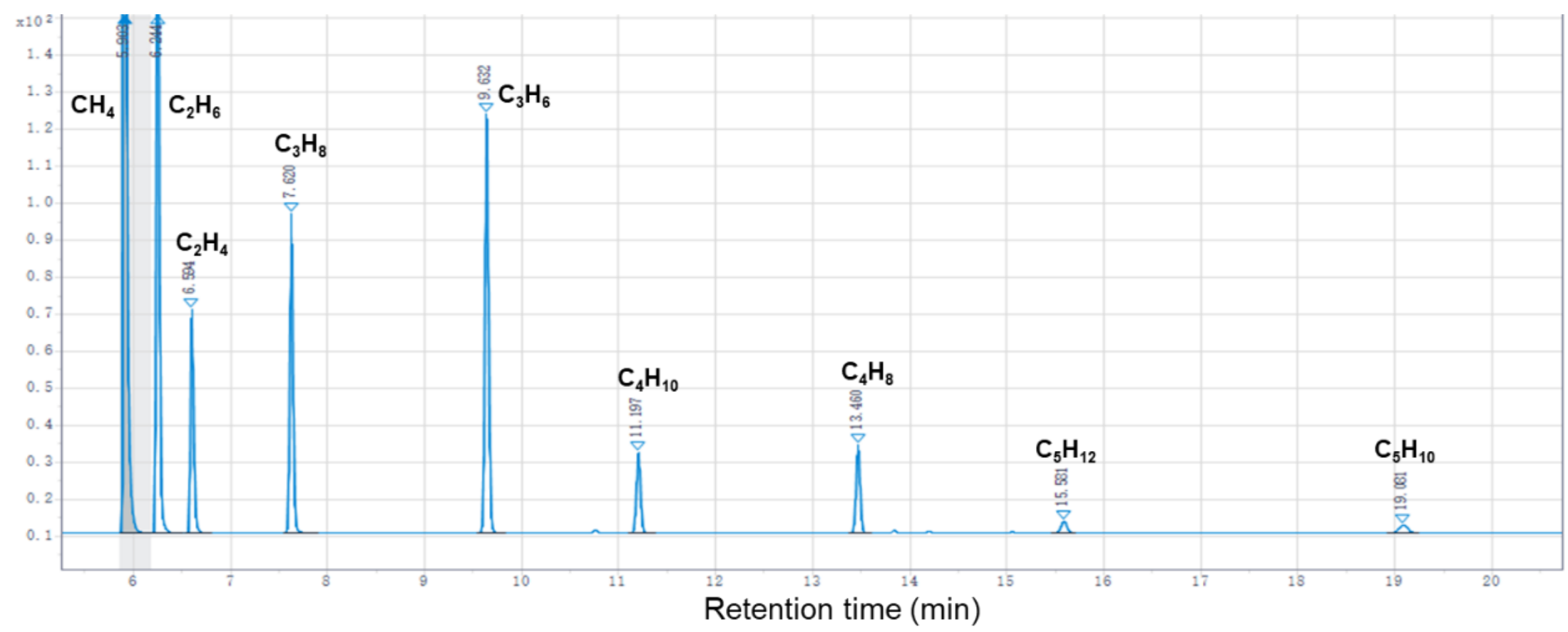

Fig. S14. The GC graph of the gaseous sample after CO_2 hydrogenation over the $Ni_{0.1}Cu_1/CoO_x$ catalyst at the optimal condition, which was obtained by a GC (Agilent 8890) instrument equipped with a flame ionization detector (FID) and a HP-plot Al_2O_3 column using N_2 as the carrier gas.

Note: The C2~C5 products in gaseous form were determined by this GC.

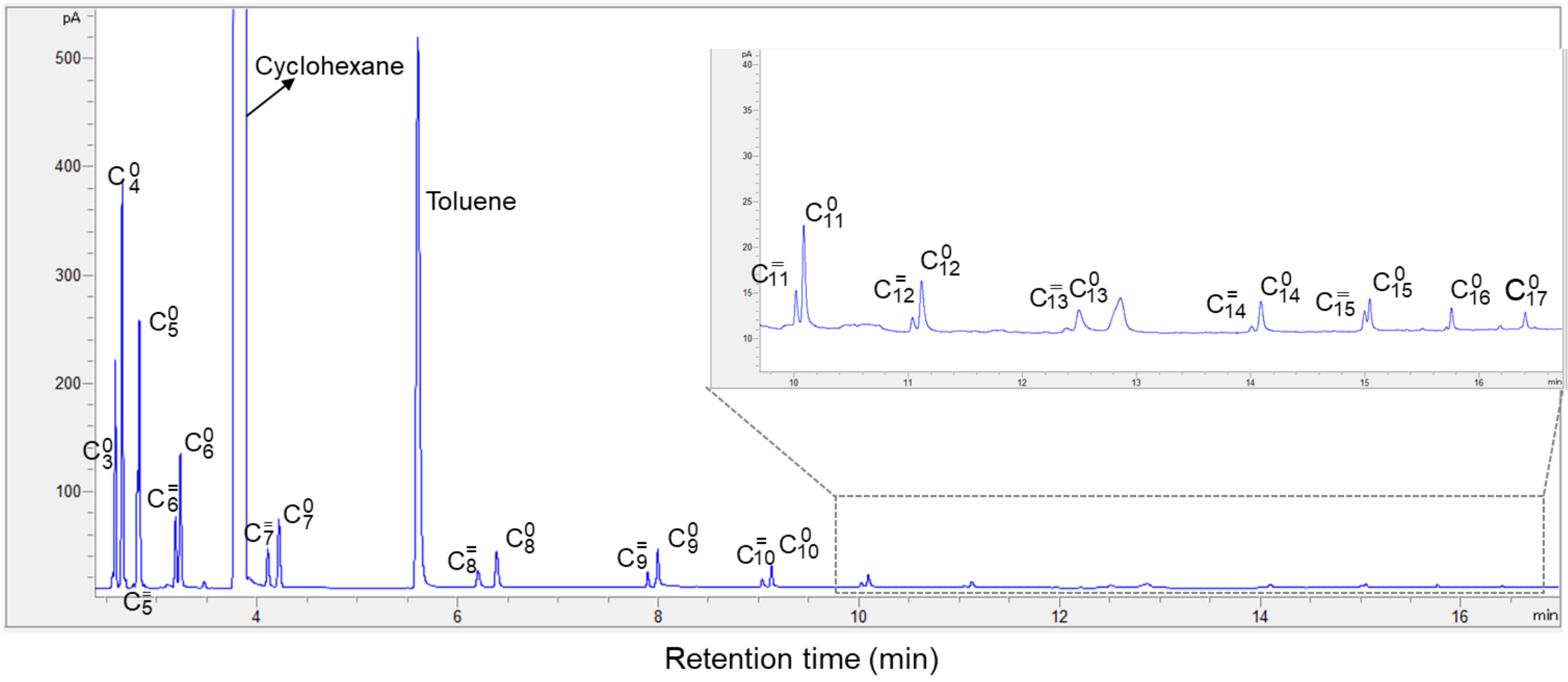

Fig. S15. The GC graph of the reaction solution after CO_2 hydrogenation over the $Ni_{0.1}Cu_1/CoO_x$ catalyst at the optimal condition, which was obtained by a gas chromatograph (GC, Agilent 7890 B) equipped with a flame ionization detector (FID) and a DB-5 capillary column using toluene as an internal standard.

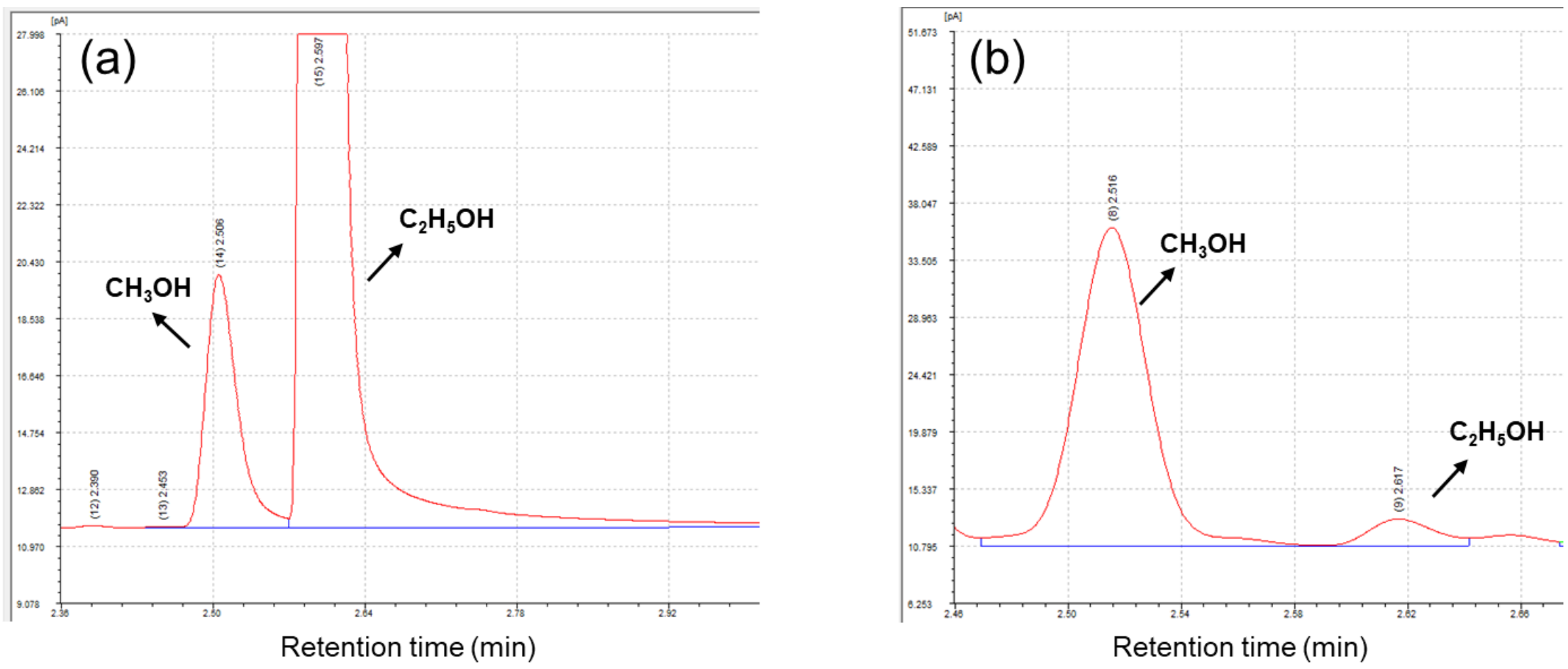

Fig. S16. GC gragh of (a) standard methanol and ethanol mixed vapor samples (b) small amounts of alcohol products after the CO_2 hydrogenation over the $Ni_{0.1}Cu_1/CoO_x$ catalyst in a stainless steel tubular fixed-bed reactor (100 mg of catalyst, 200 °C, 1 h, 4.0 MPa, $H_2/CO_2/N_2$ ratio of 48:48:4, GHSV of 100 mL min^{-1}, no solvent)

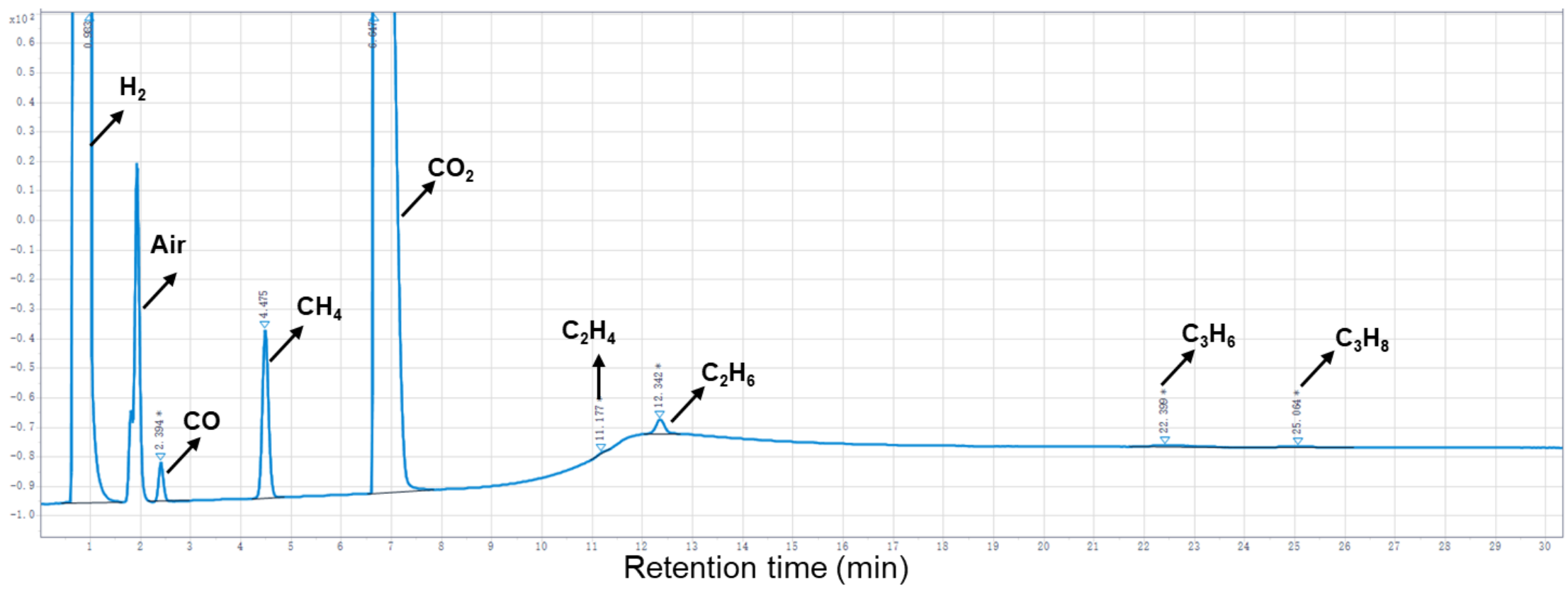

Fig. S17. The GC graph of the gaseous sample after CO_2 hydrogenation over the $Ni_{0.1}Cu_1/CoO_x$ catalyst (Reaction conditions: catalyst 50 mg and 2 mL of Cyclohexane, initial pressure 0.5 MPa (CO_2/H_2=1:1), 12 h, 200 °C), which was obtained by a gas chromatograph (GC, Agilent 7890B) equipped with a packed column (Shin Carbon ST, 2 mm in diameter and 2 m in length) and a TCD detector using argon as the carrier gas.

Note: The C1 products and CO_2 in gaseous form were determined by this GC.

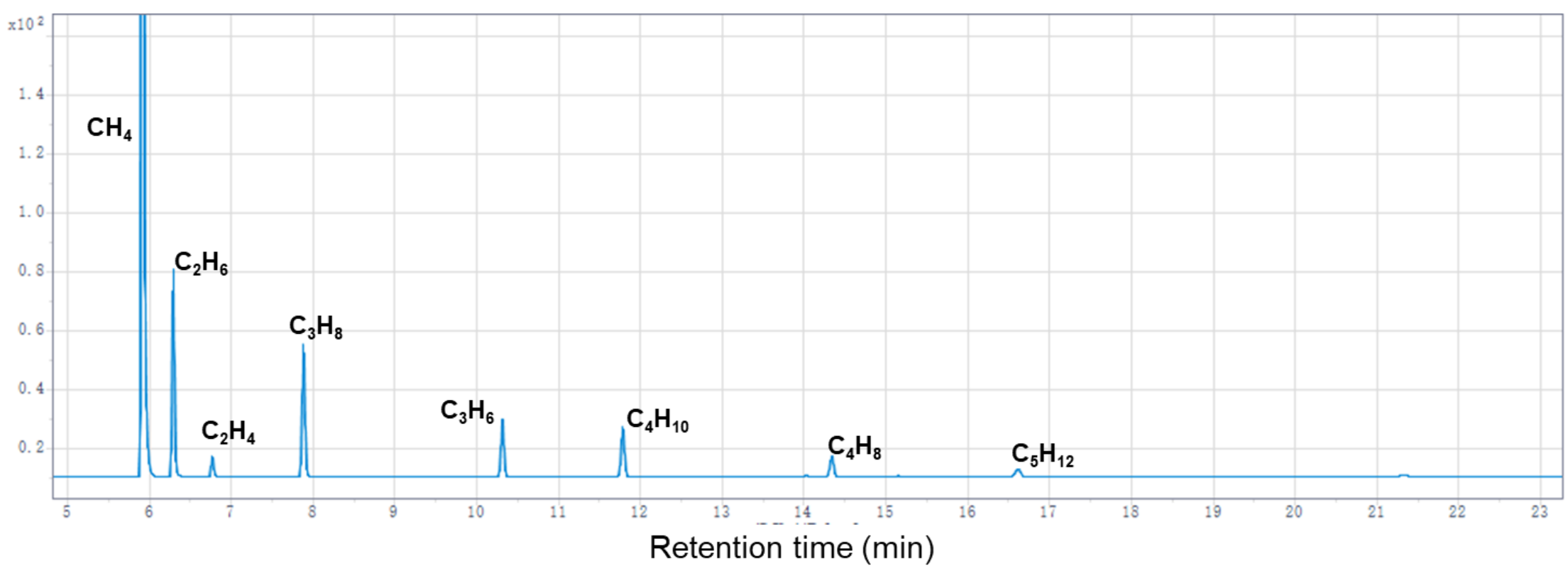

Fig. S18. The GC graph of the gaseous sample after CO_2 hydrogenation over the $Ni_{0.1}Cu_1/CoO_x$ catalyst (Reaction conditions: catalyst 50 mg and 2 mL of Cyclohexane, initial pressure 0.5 MPa (CO_2/H_2=1:1), 12 h, 200 °C), which was obtained by a GC (Agilent 8890) instrument equipped with a flame ionization detector (FID) and a HP-plot Al_2O_3 column using N_2 as the carrier gas.

Note: The C2~C5 products in gaseous form were determined by this GC.

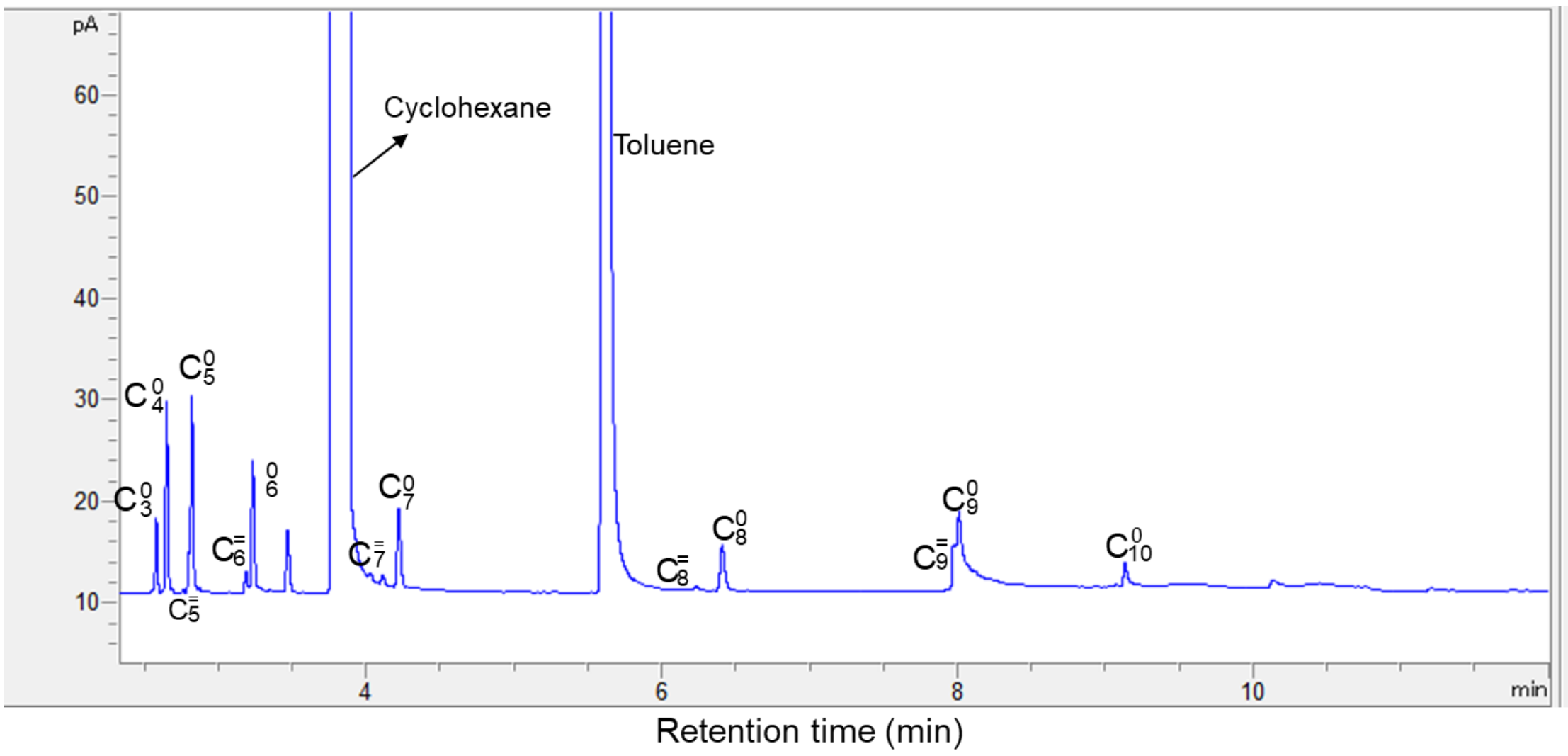

Fig. S19. The GC graph of the reaction solution after CO_2 hydrogenation over the $Ni_{0.1}Cu_1/CoO_x$ catalyst (Reaction conditions: catalyst 50 mg and 2 mL of Cyclohexane, initial pressure 0.5 MPa (CO_2/H_2=1:1), 12 h, 200 °C), which was obtained by a gas chromatograph (GC, Agilent 7890 B) equipped with a flame ionization detector (FID) and a DB-5 capillary column using toluene as an internal standard.

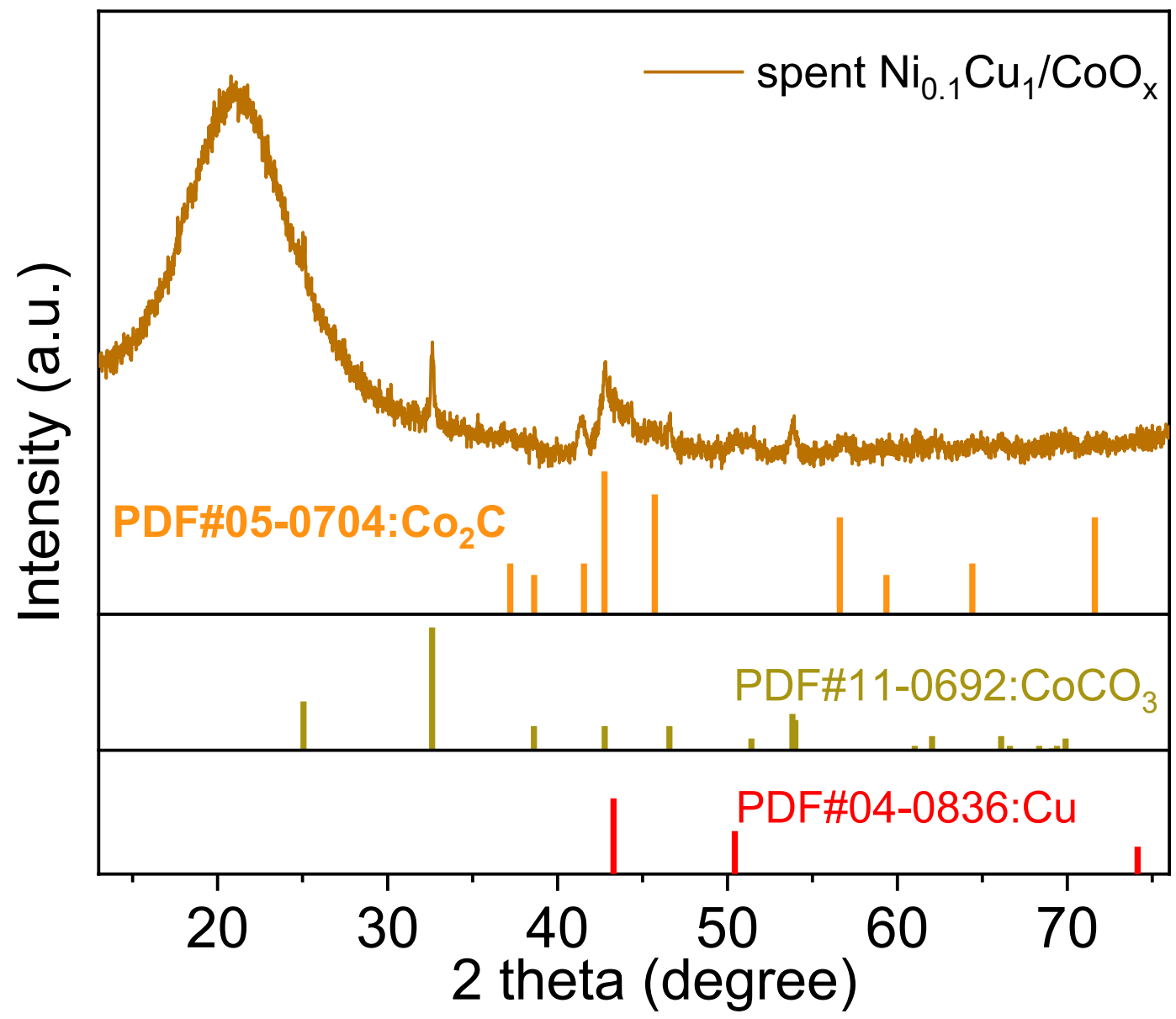

Fig. S20. XRD patterns of the spent $Ni_{0.1}Cu_1/CoO_x$ catalyst.

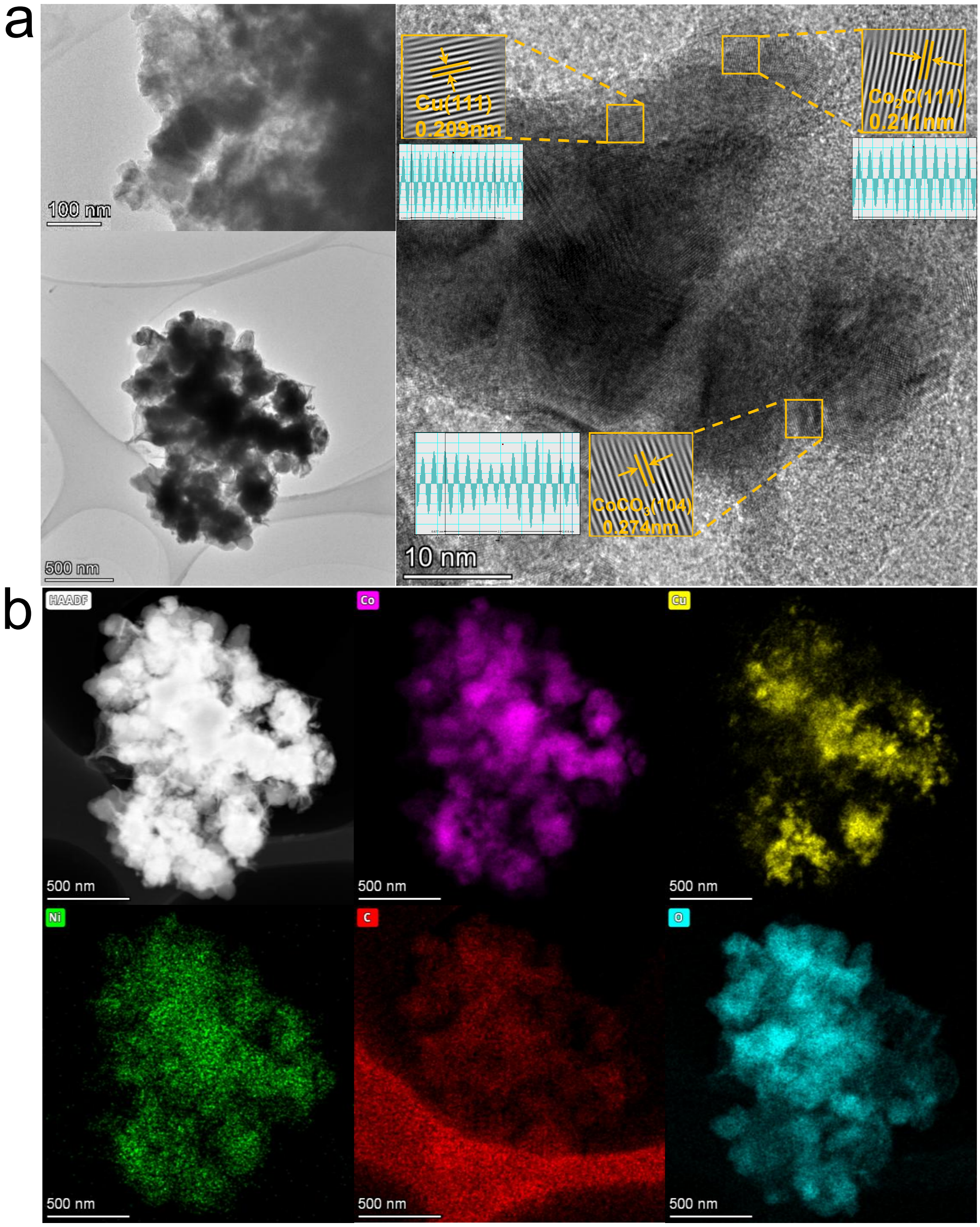

Fig. S21. (a) TEM and High Resolution(HR)HR-TEM images for spent $Ni_{0.1}Cu_1/CoO_x$ including its inverse fast Fourier transform(IFFT) images and lattice fringe distances (Insets). (b) The STEM-HAADF image and EDS mapping for Co, Cu, Ni, C, and O in the spent $Ni_{0.1}Cu_1/CoO_x$.

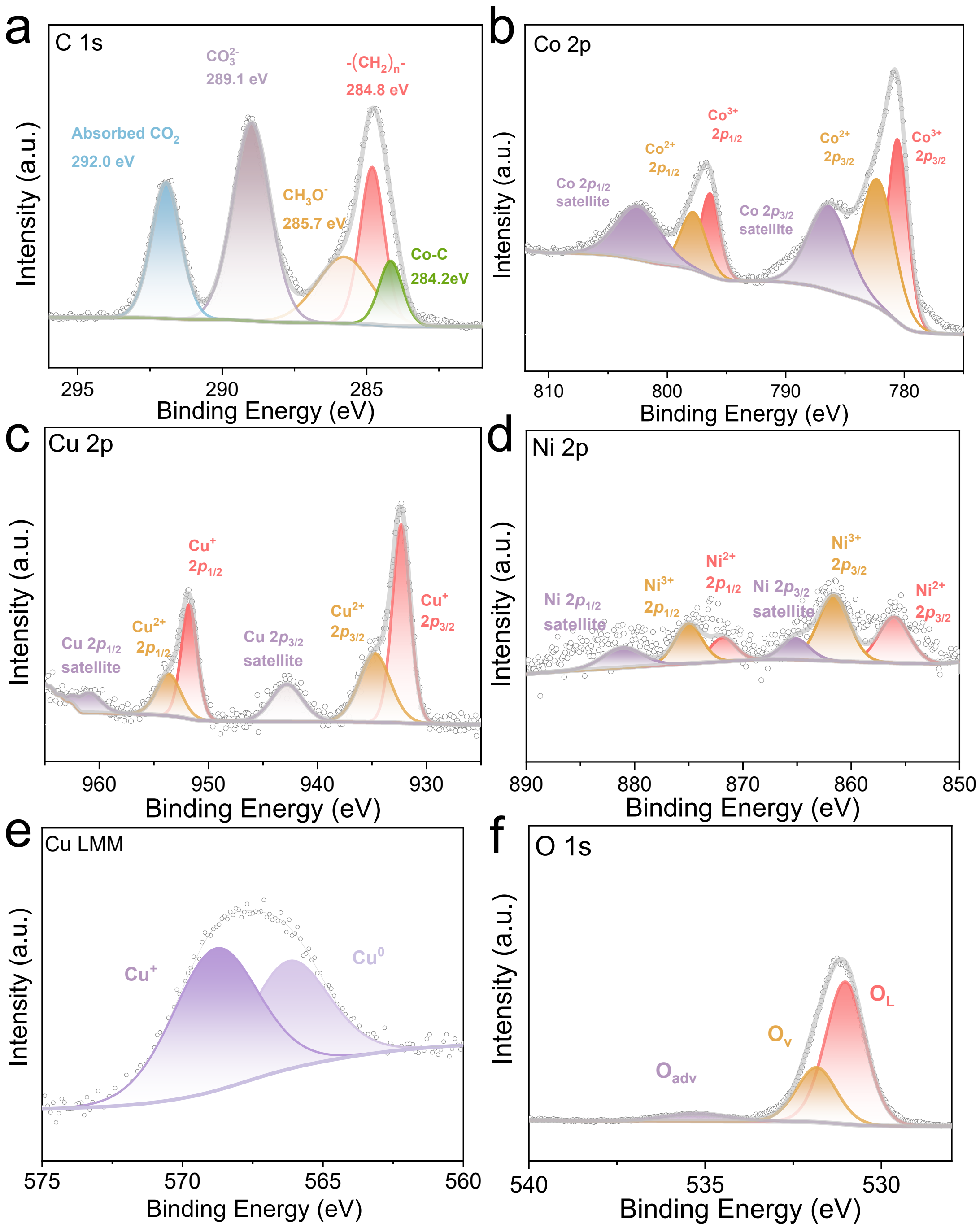

Fig. S22. The XPS spectra of the spent $Ni_{0.1}Cu_1/CoO_x$ catalyst. (a) C 1s spectra; (b) Co 2p spectra; (c) Cu 2p spectra; (d) Ni 2p spectra; (e) Cu LMM Auger spectra; (f) O 1s spectra.

Table S1 Comparison of the catalytic performance of $Ni_{0.1}Cu_1/CoO_x$ catalyst with reported heterogeneous catalysts.

Catalysts	T / °C	P / MPa	H_2/CO_2	C_{2+} HCs sel.	Reference
Fe-Co/NC-600	320	2.0	3/1	54.9	[1]
Na-Co/TiO_2	250	2.5	2/1	42.5	[2]
K-Co/SiO_2	270	0.1	3/1	30.5	[3]
K-$Co_5Cu_5O_x$	300	3.0	3/1	59.2	[4]
Na–CuCo-5	330	4.0	1/1	60.5	[5]
Na-Co/SiO_2	370	0.1	3/1	36.6	[6]
Na-Mo-Co/SiO_2	370	0.1	3/1	45.9	[6]
K-Pt-Co/SiO_2	370	0.1	3/1	37.7	[6]
CoO/TiO_2	250	2.0	3/1	10.7	[7]
Na–CuCo-9	330	4.0	1/1	45.1	[8]
$Ni_{0.1}Cu_1/CoO_x$	200	8.0	1/1	63.0	This work

Table S2 Composition and physical properties of the Ni_nCu_1/CoO_x catalysts.

Cu:Ni (1:n)	Catalysts	Ni loading[a] (wt%)	Cu loading[a] (wt%)	Co loading[a] (wt%)	Cu:Ni (actual)	S_{BET}[b] (m^2/g)	d_p[b] (nm)
1:0.1	$Ni_{0.1}Cu_1/CoO_x$	0.9	9.4	50.5	1:0.09	26.9	21.7
1:0.25	$Ni_{0.25}Cu_1/CoO_x$	1.6	7.0	38.1	1:0.23	26.6	22.4
1:0.5	$Ni_{0.5}Cu_1/CoO_x$	3.5	7.9	43.2	1:0.44	19.2	27.8
1:1	Ni_1Cu_1/CoO_x	8.1	9.0	48.5	1:0.90	18.7	43.1
1:2.5	$Ni_{2.5}Cu_1/CoO_x$	13.8	5.8	32.9	1:2.38	12.0	44.6
-	Ni/CoO_x	9.3	-	46.5	-	30.9	17.5
-	Cu/CoO_x	-	14.9	54.3	-	13.7	35.8
-	Ni/CuO_x	35.6	30.7	-	-	8.9	22.5
-	After calcination[c]	-	-	-	-	17.8	35.3
-	Before calcination[c]	-	-	-	-	67.5	10.4

a Determined by ICP-OES.

b The surface areas (S_{BET}) and average pore diameters (d_p) were measured by N_2 adsorption-desorption test.

c The precipitate before and after the calcination during the preparation $Ni_{0.1}Cu_1/CoO_x$ catalyst.

Table S3 Surface-area-normalized activity of the Cu/CoO_x and $Ni_{0.1}Cu_1/CoO_x$ catalysts.

Catalysts	S_{BET} (m^2/g)	d_p (nm)	Surface-area-normalized activity (C_{2+}) (mmol m^{-2} h^{-1})
$Ni_{0.1}Cu_1/CoO_x$	26.9	21.7	0.055
Cu/CoO_x	13.7	35.8	0.080

Note: The surface areas (S_{BET}) and average pore diameters (d_p) were measured by N_2 adsorption-desorption test.

References

1. Satthawong, R.; Koizumi, N.; Song, C.; Prasassarakich, P. Bimetallic Fe–Co catalysts for CO_2 hydrogenation to higher hydrocarbons. *J. CO2 Util.* **2013,** *3-4*, 102-106.

2. Scarfiello, C.; Soulantica, K.; Cayez, S.; Durupt, A.; Viau, G.; Le Breton, N.; Boudalis, A. K.; Meunier, F.; et al. Modified Co/TiO_2 catalysts for CO_2 hydrogenation to fuels. *J. Catal.* **2023,** *428*, 115202.

3. Iloy, R. A.; Jalama, K. Effect of Operating Temperature, Pressure and Potassium Loading on the Performance of Silica-Supported Cobalt Catalyst in CO_2 Hydrogenation to Hydrocarbon Fuel. *Catalysts* **2019,** *9* (10), 807.

4. Wang, M.; Guo, S.; Yan, S.; Wang, Z.; Zhang, G.; Gao, H.; Zhang, M.; Bian, K.; Liu, J.; Nie, X.; et al. Engineering the Cu(0)–Co_2C Interface via Reaction-Induced Reconstruction for CO_2 Hydrogenation to C_{2+} Hydrocarbons. *J. Am. Chem. Soc.* **2025,** *147* (45), 42051-42060.

5. Irshad, M.; Jo, H.; Bibi, S. S.; Kim, J. Synthesis of selective C_{5+} paraffins directly from CO_2 over Cu-Co catalyst: Dynamic role of Cu to establish the Fischer–Tropsch synthesis process. *Chem. Eng. J.* **2025,** *517*, 164291.

6. Owen, R. E.; O'Byrne, J. P.; Mattia, D.; Plucinski, P.; Pascu, S. I.; Jones, M. D. Cobalt catalysts for the conversion of CO_2 to light hydrocarbons at atmospheric pressure. *Chem. Commun.* **2013,** *49* (99), 11683-11685.

7. Have, I. C. t.; Kromwijk, J. J. G.; Monai, M.; Ferri, D.; Sterk, E. B.; Meirer, F.; Weckhuysen, B. M. Uncovering the reaction mechanism behind CoO as active phase for CO_2 hydrogenation. *Nat. Commun.* **2022,** *13* (1), 324.

8. Irshad, M.; Chun, H.-J.; Khan, M. K.; Jo, H.; Kim, S. K.; Kim, J. Synthesis of n-butanol-rich C_{3+} alcohols by direct CO_2 hydrogenation over a stable Cu–Co tandem catalyst. *Appl. Catal. B: Environ.* **2024**, *340*, 123201.
